# Polarisation effects on the solvation properties of alcohols†
†Electronic supplementary information (ESI) available: Experimental and computational details. See DOI: 10.1039/c7sc04890d


**DOI:** 10.1039/c7sc04890d

**Published:** 2017-12-06

**Authors:** Stefan Henkel, Maria Cristina Misuraca, Pavle Troselj, Jonathan Davidson, Christopher A. Hunter

**Affiliations:** a Department of Chemistry , University of Cambridge , Lensfield Road , Cambridge CB2 1EW , UK . Email: herchelsmith.orgchem@ch.cam.ac.uk

## Abstract

Alcohol solvents are significantly more polar than expected based on the measured H-bonding properties of monomeric alcohols in dilute solution. Self-association of alcohols leads to formation of cyclic aggregates and linear polymeric chains that have a different polarity from the alcohol monomer. Cyclic aggregates are less polar than the monomer, and the chain ends of linear polymers are more polar. The solvation properties of alcohols therefore depend on the interplay of these self-association equilibria and the equilibria involving interactions with solutes. Twenty-one different molecular recognition probes of varying polarity were used to probe the solvation properties of alkane–alcohol mixtures across a wide range of different solvent compositions. The results allow dissection of the complex equilibria present in these systems. Formation of a H-bond between two alcohol molecules leads to polarisation of the hydroxyl groups, resulting in an increase in binding affinity for subsequent interactions with the unbound donor and acceptor sites. The H-bond donor parameter (*α*) for these sites increases from 2.7 to 3.5, and the H-bond acceptor parameter (*β*) increases from 5.3 to 6.9. Polarisation is a short range effect limited to the first H-bond in a chain, and formation of subsequent H-bonds in longer chains does not further increase the polarity of chain ends. H-bond donor sites involved in a H-bond are unavailable for further interactions, because the formation of a bifurcated three-centre H-bond is three orders of magnitude less favourable than formation of a conventional two-centre H-bond. These findings are reproduced by quantum chemical calculations of the molecular electrostatic potential surfaces of alcohol aggregates. Thus, the overall solvation properties of alcohols depend on the speciation of different aggregates, the polarities of these species and the polarities of the solutes. At low alcohol concentrations, polar solutes are solvated by alcohol monomers, and at higher alcohol concentrations, solutes are solvated by the more polar chain ends of linear polymers. The less polar cyclic aggregates are less important for interactions with solutes. Similar behavior was found for ten different alcohol solvents. Tertiary alcohols are marginally less polar solvents than primary alcohols, due to steric interactions that destabilises the formation of polymeric aggregates leading to lower concentrations of polar chain ends. One alcohol with an electron-withdrawing substituent was studied, and this solvent showed slightly different behavior, because the H-bond donor and acceptor properties are different.

## Abstract 2

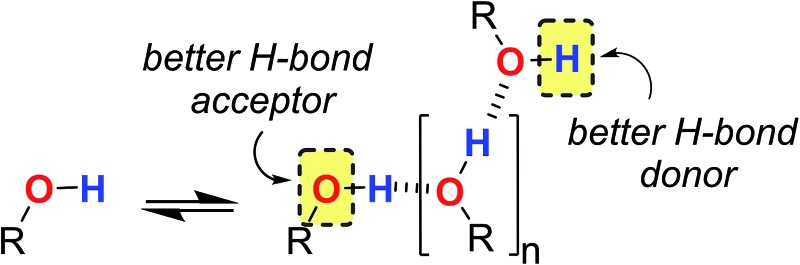
Alcohol solvents are significantly more polar than expected based on the measured H-bonding properties of monomeric alcohols in dilute solution.

## Introduction

**Fig. 1:**
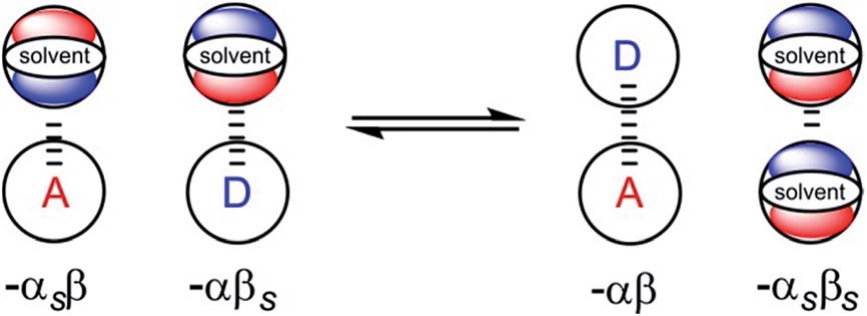
Schematic representation of the electrostatic solvent competition model. The free energy of interaction between acceptor (A) and donor (D) solutes can be estimated from the H-bond parameters of the solutes (*α*, *β*) and of the solvent (*α*_S_, *β*_S_) according to eqn (1).

From industrial to biomolecular processes, solvation is a fundamental aspect of every chemical reaction that takes place in solution. Quantitative description of a solution at the molecular level is challenging due to the large number of species and interactions present in the liquid phase. Empirical models are useful tools for describing solutions, because they can provide estimates of the thermodynamic properties of non-covalent interactions between solvents and solutes using molecular or functional group parameters that have been obtained experimentally.^1^,^2^ By treating solutions as pairwise interactions between H-bond donor and acceptor sites (Fig. 1), the relationship embodied in eqn (1) is obtained. This expression provides an estimate of the free energy of any non-covalent interaction between two solutes in any solvent provided the H-bond parameters *α* and *β* are available.^3^

1 Δ*G*/kJ mol^–1^ = –*RT* ln *K* = –(*α* – *α*_S_)(*β* – *β*_S_) + 6

In this model, the H-bond donor and acceptor parameters for the solutes and for the solvents are taken from to the same H-bond scales, which were derived from empirical solute parameters. These scales have been further extended using calculations of molecular electrostatic potential surfaces. A central assumption of this model is that the bulk solvent molecules behave in the same way as isolated solvent molecules in a dilute solution with respect to intermolecular interactions, *i.e.* the solute and solvent molecules can be treated in exactly the same way.

This assumption is supported by the presence of (i) a correlation between the empirical H-bond parameters for bulk solvents introduced by Taft and the corresponding parameters describing the H-bond properties of solutes derived by Abraham and others^4^,^5^ and (ii) a correlation between the empirical parameters *α* and *β* and the minima and maxima of the molecular electrostatic potential surfaces calculated in the gas phase.^3^,^6^ The validity of this approach has been shown by the quantitative applicability of eqn (1) to estimate the stability of various H-bonded complexes in a wide range of different solvents and, notably, also in solvent mixtures.^7^–^11^ The model is not limited to H-bonded systems, but it describes other kinds of non-covalent interactions, such as halogen-bonding and aromatic interactions.^12^–^16^

The model embodied in eqn (1) has been extended in order to take into account all intermolecular interactions of a molecule with its solvation shell, rather than just the strongest one.^6^,^17^ In this approach, a molecule is described as a set of surface site interaction points (SSIPs) that allow treatment of molecules with multiple functional groups. The pairwise interaction of any two SSIPs can be estimated based on the H-bond parameters, giving a comprehensive description of the interactions present in liquid mixtures.

**Fig. 2:**
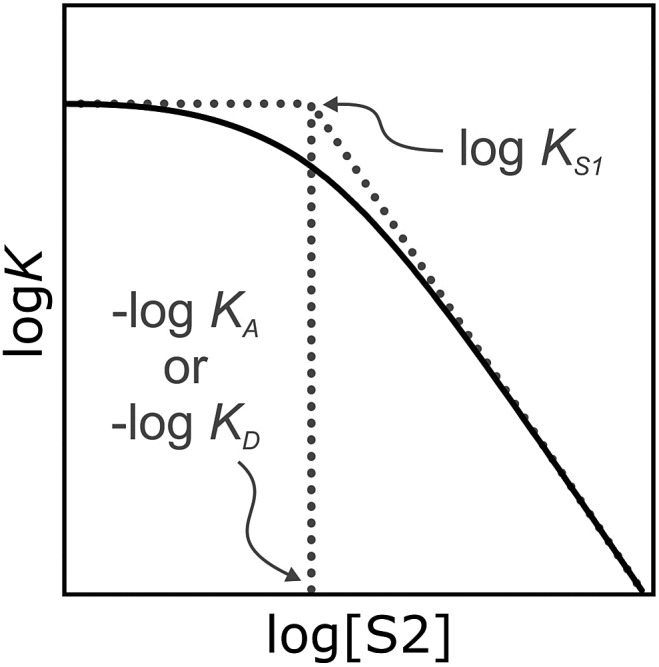
Dependence of the association constant (log *K*) for formation of a 1 : 1 complex between a H-bond donor and acceptor on the concentration of a polar solvent (S2) in a non-polar solvent (S1). log *K*_S1_ is the D·A association constant in pure S1, and log *K*_D_ and log *K*_A_ are the D·S2 and A·S2 association constants in pure S1.

It has been noted that solvents that self-associate to a significant extent, such as alcohols, are not described properly by this model. For such solvents, H-bond parameters derived from molecular properties are not sufficient to account for the behavior of the bulk solution.^4^,^5^,^18^ In order to dissect the equilibria that determine the solvation properties of self-associating solvents, a series of experiments was carried out using a molecular recognition probe in mixtures of a self-associating polar solvent and a non-polar co-solvent. Typical results obtained from such a mixed solvent study are illustrated in Fig. 2. The association constant (*K*) for formation of a 1 : 1 complex between a H-bond donor (D) and a H-bond acceptor (A) is measured as a function of the concentration of a polar solvent (S2) in a non-polar solvent (S1). Fig. 2 shows that at low concentrations of S2, log *K* remains constant at the value measured in pure S1 (log *K*_S1_). When the concentration of S2 is high enough for preferential solvation of one of the two solutes, the value of log *K* drops due to competition with solvation by the polar solvent S2. If D interacts more strongly with S2 than A, the onset of the drop in log *K* occurs at a concentration of S2 that depends on the value of *K*_D_, the association constant for formation of the D·S2 complex in S1. Similarly, if A interacts more strongly with S2, the change in log *K* occurs at log[S2] = –log *K*_A_, where *K*_A_ is the association constant for formation of the A·S2 complex in S1. For solvents that have one polar site that dominates interactions with solutes, the behavior illustrated in Fig. 2 is described well by eqn (2).2
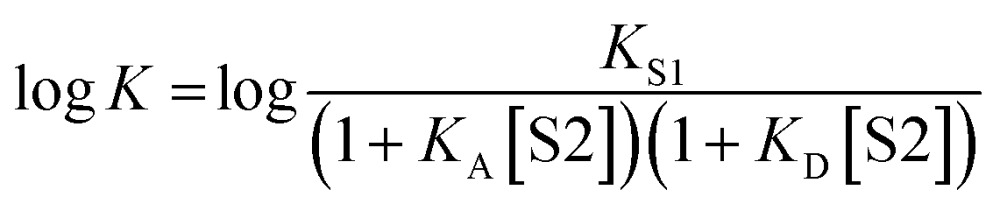



**Fig. 3:**
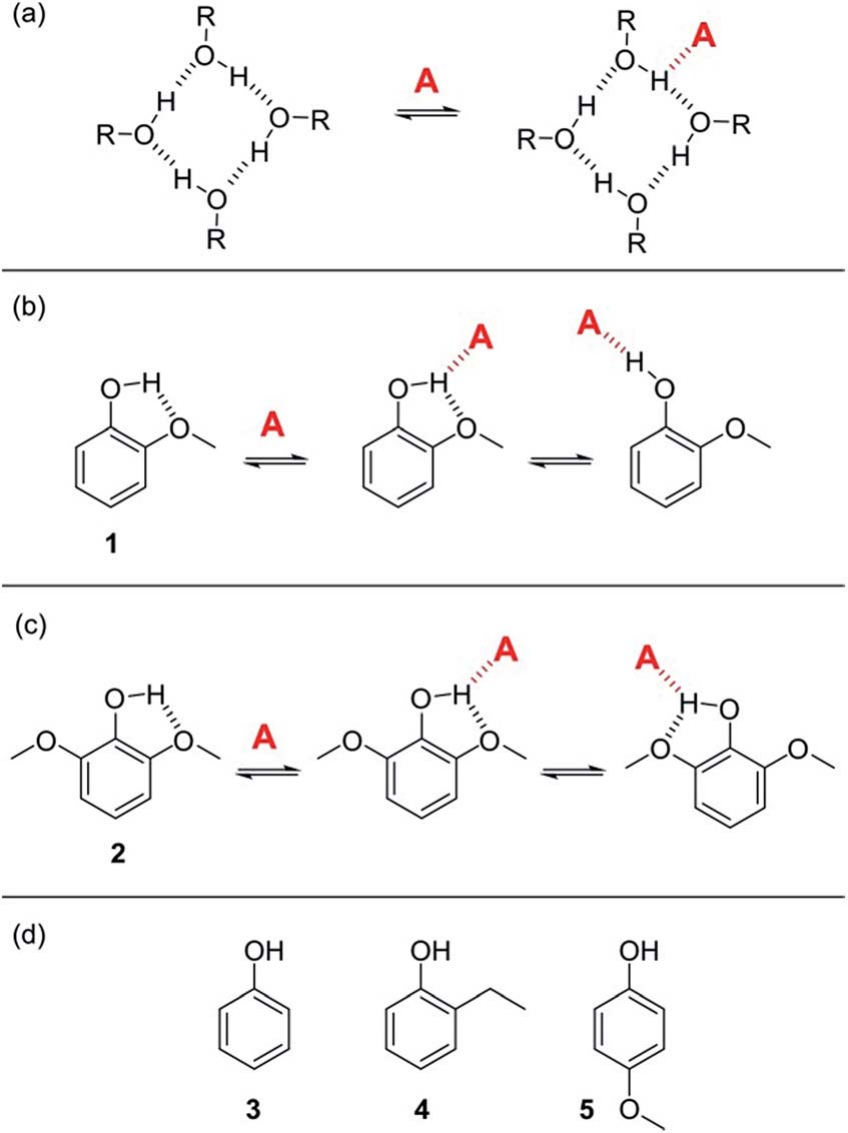
(a) Proposed bifurcated H-bond formed between an alcohol aggregate and a H-bond acceptor (A).^8^ (b) The two possible binding modes of **1** with a H-bond acceptor. Breaking the intramolecular H-bond in the three-site bifurcated H-bond would allow formation of a conventional two-site H-bond. (c) The two possible binding modes of **2** with a H-bond acceptor (A) both involve a bifurcated H-bond. (d) Reference phenols **3–5** that do not make intramolecular H-bonds.

For solvents that feature only H-bond acceptor properties, *K*_A_ is small and the decrease in log *K* is only due to solvation of the donor D. Likewise, solvents that primarily have H-bond donor properties will only solvate acceptor A. In these cases, the slope of the log *K vs.* log[S2] profile in the high [S2] regime shown in Fig. 2 is –1, as has been demonstrated for a wide range of solvents.^10^ For solvents that are both H-bond donors and acceptors, the behavior is more complicated, because both solutes D and A can be preferentially solvated by S2 and these solvation equilibria are in competition with self-association of S2. When 1-octanol was used as S2 in mixtures with *n*-octane as S1, the slope of the log *K vs.* log[S2] profile in the high [S2] regime was found to be –2. This result could be rationalised if alcohols maintain their H-bond donor properties at concentrations where aggregates are the dominant species, *i.e.* self-association does not affect the solvation properties.^19^ Specifically, it was possible to account for the slope of –2 by assuming that the association constant for the formation of a bifurcated H-bond between an alcohol aggregate and the H-bond acceptor solute (A) is similar to the association constant for the formation of a H-bond between a monomeric alcohol and solute A (Fig. 3a).

Here we show that this hypothesis is incorrect and that three-site bifurcated H-bonds are significantly weaker than simple two-site H-bonding interactions. An alternative explanation for the unusual solvation properties of alcohol solvents is therefore required. This paper reports a more detailed study of alcohol solvation in mixtures, using a diverse set of different molecular recognition probes and a variety of different alcohols. The results allow quantitative dissection of all of the competing equilibria and show that self-association of alcohols affects both the concentrations and the polarities of the functional groups available to interact to with solutes. We show that polarisation of the alcohol hydroxyl group in H-bonded aggregates holds the key to understanding the solvation properties of these solvents.

## Results

### Bifurcated H-bonds

In order to quantify the strength of bifurcated H-bonding interactions, we investigated the H-bond donor properties of hydroxyl protons that are already involved in an intramolecular H-bonding interaction. Phenols that have methoxy substituents in the *ortho*-position form intramolecular H-bonds in the free state and are good candidates for characterising bifurcated H-bonds. Fig. 3b shows that phenol **1** could bind to a H-bond acceptor by forming a bifurcated three-site H-bond. However, a conventional two-site H-bond could also be formed by breaking the intramolecular H-bond. The observed association constant would be the sum of the association constants for formation of these two different complexes. In phenol **2**, the hydroxyl group is flanked on both sides by a methoxy substituent (Fig. 3c), so it would only be possible to break the intramolecular H-bond by twisting the hydroxyl group orthogonal to the aromatic ring, which would not only break the conjugation of the oxygen lone pair with the ring, it would also dramatically reduce the H-bond donor parameter: *α* = 3.8 for phenol compared with *α* = 2.7 for an alkyl alcohol. Measurement of the H-bond donor properties of **1** and **2** should therefore provide an experimental test of the bifurcated H-bond hypothesis used previously to rationalise the solvation properties of alcohols.^8^

The formation of 1 : 1 complexes of phenols **1–5** with tri *n*-butyl phosphine oxide **6** in *n*-octane was investigated by UV-vis absorption and ^1^H NMR titrations. Phenols **3–5** are control compounds that do not form intramolecular H-bonds, but provide an indication of the steric effect of an *ortho* substituent and the electronic effect of a methoxy substituent on the ring (Fig. 3d). The association constants for phenols **3–5** shown in Table 1 indicate that the steric and electronic effects of the substituents are not significant. However, the association constants for the phenols that form intramolecular H-bonds are substantially lower than for the phenols that do not. The **1·6** complex is slightly more stable than the **2·6** complex, which is probably due to population of the state in which the intramolecular H-bond is broken in the **1·6** complex. Comparison of the association constant for the complexes formed with **1** and **2** with the values for phenols **3–5** suggests that the bifurcated H-bonds are about three orders of magnitude less stable than the conventional H-bonds.^20^,^21^ This result implies that the unusual solvation properties of alcohols reported previously cannot be rationalised on the basis of bifurcated H-bonding interactions with alcohol aggregates.^8^

**Table 1:** Association constants (log *K*/M^–1^) for formation of 1 : 1 complexes with **6** in *n*-octane at 298 K^*a*^

	**1**	**2**	**3**	**4**	**5**
log *K*/M^–1^	1.7	1.2	4.2	3.9	3.9

^*a*^Associations constants were determined by UV-vis titrations, and for complexes **1·6** and **4·6** NMR titrations were also carried out.

### Effect of solute polarity on solvation

**Fig. 4:**
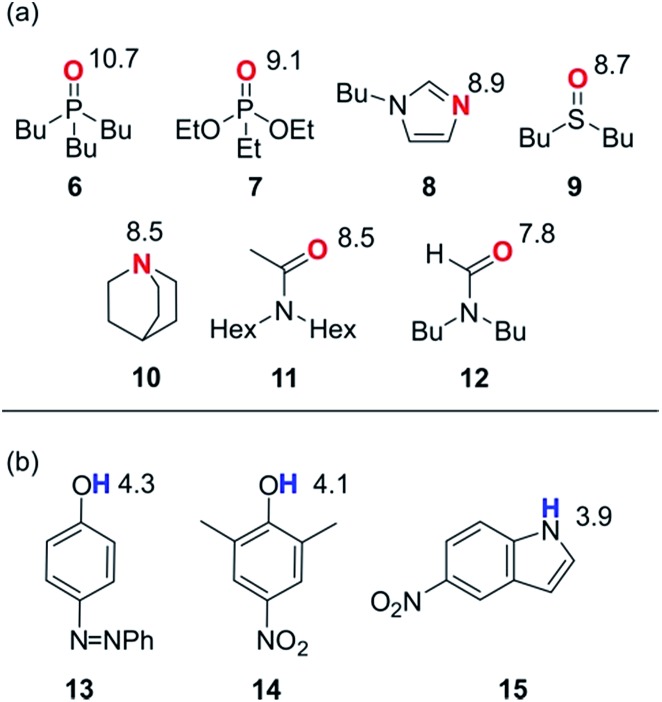
Molecular recognition probes with values of the H-bond parameters, *β* and *α*. (a) H-bond acceptors (*β*). (b) H-bond donors (*α*).

In order to probe the solvation properties of alcohols in more detail, we therefore investigated the effects of alcohol solvation on a set of molecular recognition probes of differing polarity. Tri-*n*-butyl phosphine oxide and 4-phenyl azophenol were used previously, because these solutes form a strongly H-bonded complex and the azophenol provides convenient spectroscopic probe for UV-vis titrations.^8^ The H-bond acceptors and donors shown in Fig. 4 can be used to form 21 different complexes that can also be monitored using UV-vis spectroscopy. The association constants for all of complexes were measured in *n*-octane (log *K*_S1_), and the results are reported in Table 2. The experimental values span over three orders of magnitude and are in agreement with values estimated using the H-bond parameters shown in Fig. 4.

**Table 2:** Association constants (log *K*_S1_/M^–1^) for formation of 1 : 1 complexes in *n*-octane at 298 K

		H-bond acceptors						
		**6**	**7**	**8**	**9**	**10**	**11**	**12**
H-bond donors	**13**	5.0	3.8	3.7	3.7	3.4	3.4	2.9
	**14**	4.5	3.4	3.4	3.3	2.9	3.1	2.6
	**15**	4.1	3.1	3.0	3.0	2.4	2.8	2.3

**Fig. 5:**
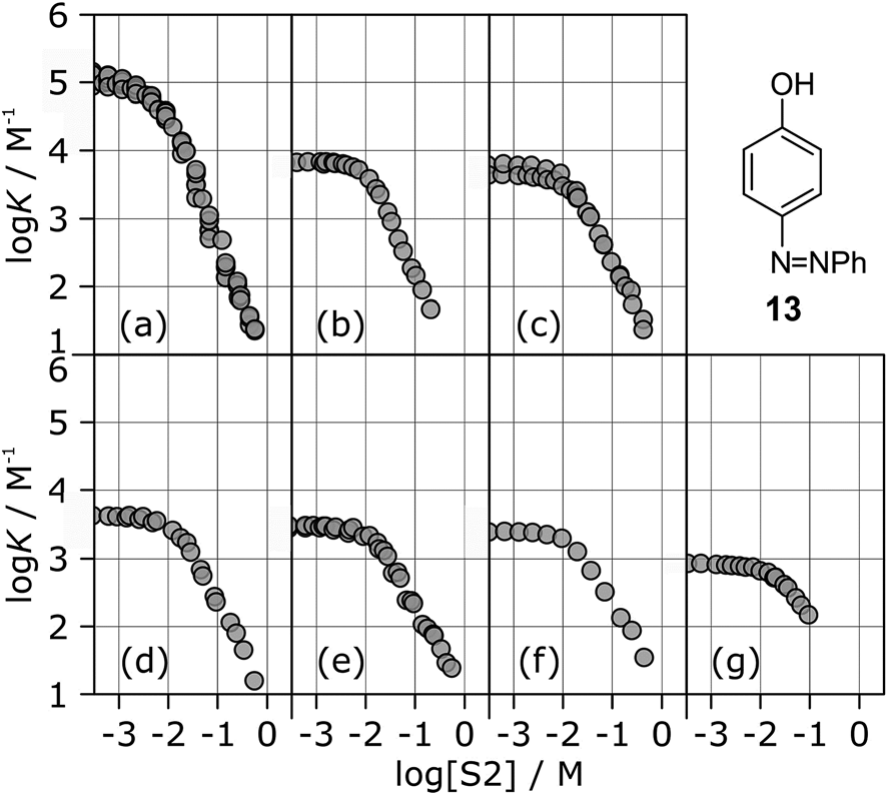
Association constants (log *K*/M^–1^) for formation of 1 : 1 complexes between H-bond donor **13** and different H-bond acceptors as a function of concentration of 1-octanol (S2) in *n*-octane (S1) at 298 K. Acceptors are (a) **6**, (b) **7**, (c) **8**, (d) **9**, (e) **10**, (f) **11**, (g) **12**.

The stabilities of these complexes were then investigated in mixtures of *n*-octane (S1) and 1-octanol (S2) using automated UV-vis titrations. The association constants measured for the complexes formed between all of the H-bond acceptors (**6–12**) and H-bond donor **13** are shown in Fig. 5. The relationships between log *K* and log[S2] are the same as illustrated in Fig. 2. At low [S2], the values of log *K* are constant and equal to the value of log *K*_S1_ in Table 2. Once sufficient S2 has been added, the association constants decrease with increasing [S2].

The differences between the log *K vs.* log[S2] profiles in Fig. 5 are related to differences in the H-bond acceptor properties of the solutes. The value of log *K* in the constant regime at low [S2] (log *K*_S1_) decreases with decreasing H-bond acceptor strength, and the value of log[S2] at which solvation of the acceptor by the alcohol begins to compete with complexation (–log *K*_A_) also decreases with decreasing H-bond acceptor strength. For the weaker complexes, the value of log[S2] at which the value of log *K* begins to drop reaches a limiting value of –2, which corresponds to the S2 concentration at which the H-bond donor **13** becomes solvated by S2 (log *K*_D_). At high [S2], the slopes of the log *K vs.* log[S2] profiles in Fig. 5 also depend on the solute, ranging from about –2 for the strongest H-bond acceptor, **6**, to about –1 for the weakest acceptor, **12**. Table 3 gives the values of the slope of the log *K vs.* log[S2] profile in the high [S2] regime for all of the H-bond donor–acceptor combinations studied.

**Table 3:** Slopes of the log *K vs.* log[S2] profiles in the high [S2] regime

		H-bond acceptors						
		**6**	**7**	**8**	**9**	**10**	**11**	**12**
H-bond donors	**13**	–1.8	–1.3	–1.3	–1.3	–1.2	–1.1	–0.9
	**14**	–1.4	–1.0	^*a*^	^*a*^	^*a*^	^*a*^	^*a*^
	**15**	–1.3	–0.9	^*a*^	^*a*^	^*a*^	^*a*^	^*a*^

^*a*^Slopes for the complexes formed between **8–12** and **14,15** could not be determined reliably.

**Fig. 6:**
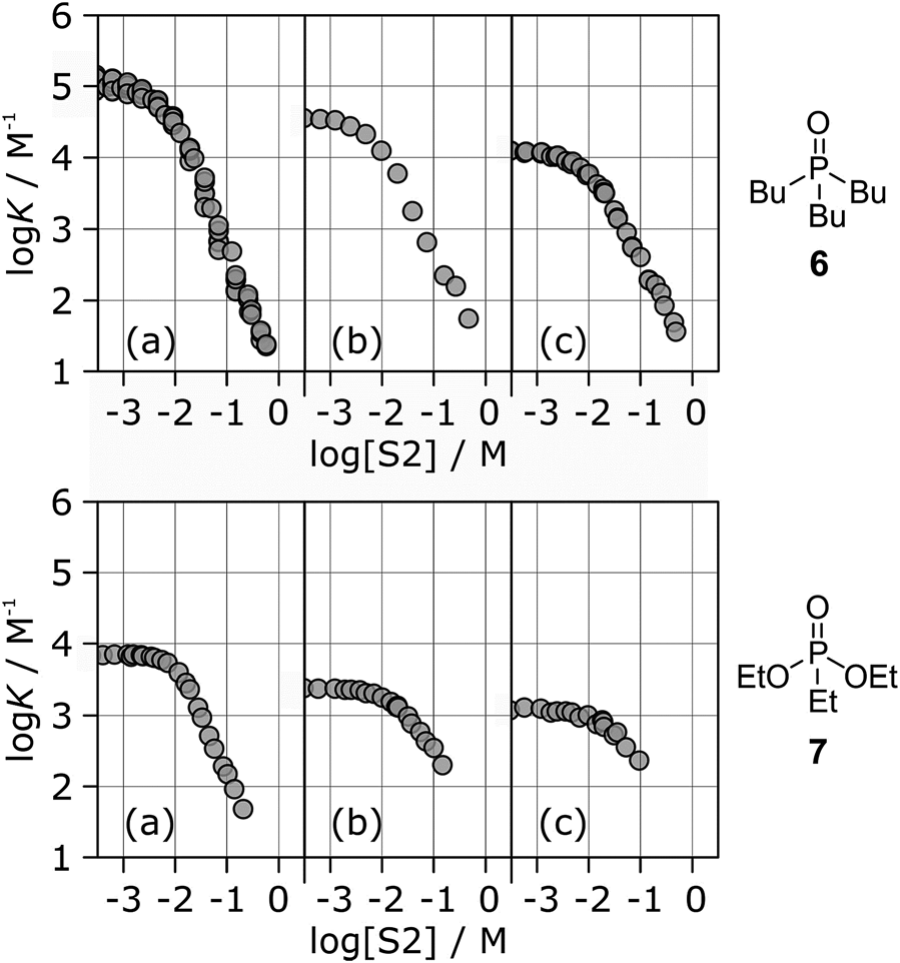
Association constants (log *K*/M^–1^) for formation of 1 : 1 complexes between H-bond acceptor **6** (upper row) or **7** (lower row) and different H-bond donors as a function of concentration of 1-octanol (S2) in *n*-octane (S1) at 298 K. Donors are (a) **13**, (b) **14**, (c) **15**.

Fig. 6 shows the association constants for the complexes formed between acceptors **6** and **7** and the three different H-bond donors **13–15** in mixtures of *n*-octane and 1-octanol. The variation in the log *K vs.* log[S2] profiles with H-bond donor properties is similar to that observed for the H-bond acceptors in Fig. 5. For the strongest H-bond donor, the association constant in the low [S2] regime is higher, the concentration of S2 at which solvation of the donor by the alcohol begins to compete with complexation is lower, and the slope in the high [S2] regime is more negative (Table 3).

These experiments show that the slope of approximately –2 that was previously reported for the high [S2] regime of the log *K vs.* log[S2] profile for the **6·13** complex is not a general property of the alcohol solvent. The value of the slope also depends on the nature of the solutes. For less polar solutes, the slope is –1, which is the same as the value observed in mixtures of alkanes and polar solvents that do not self-associate. These results indicate that there is an interplay between solvent self-association and solute polarity that leads to qualitative differences between the nature of the solvation shells in these systems.

### Speciation of alcohol aggregates

**Fig. 7:**
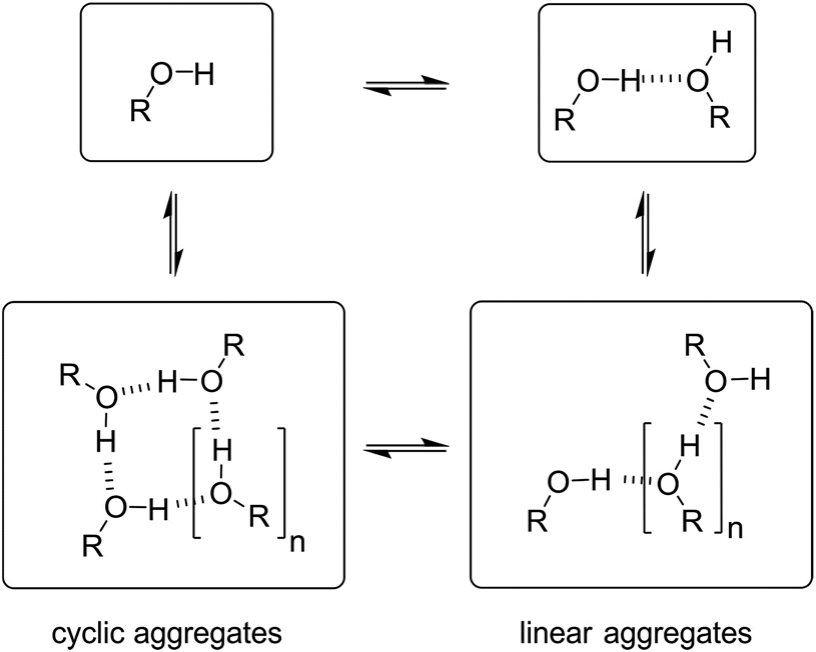
Self-association of alcohols into dimers, linear and cyclic aggregates.

In order to rationalise the influence of alcohol self-association on the experimental data described above, the concentration dependence of the speciation of different aggregated states of alcohols is required. Self-association of alcohols in alkane solution has been studied by various methods, and it is well established that both linear and cyclic assemblies are formed (Fig. 7). The relative amounts of different species present in an alcohol solution depend on the nature and concentration of the alcohol as well as the non-polar co-solvent.

For primary alcohols, simple models including only cyclic tetrameric species,^22^ as well as more sophisticated models including linear and cyclic aggregates without size-limitation,^23^ have been described. Polymerisation of alcohols into linear chains can be related to the concentration of monomeric alcohol [m] by eqn. (3) and (4).^24^ The concentrations of internal [i] and terminal [t] donor and acceptor groups present in linear chains is given by:3
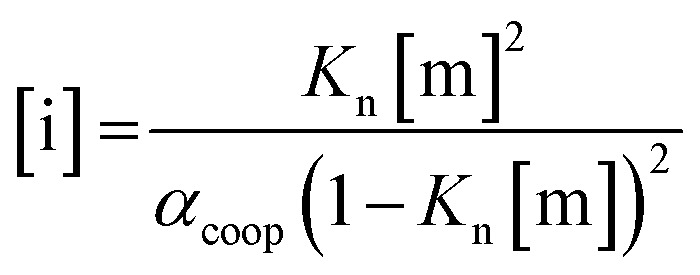

4
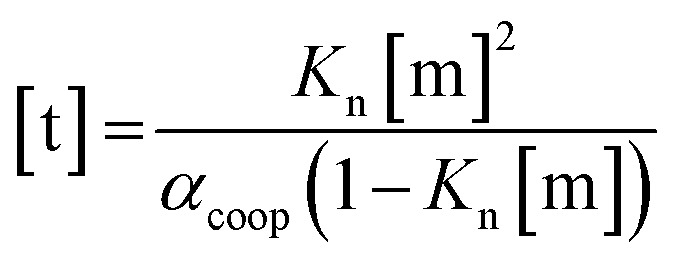
where *α*_coop_ is the cooperativity factor that describes the deviation from an isodesmic polymerisation isotherm, *K*_n_/*α*_coop_ is the association constant for the first H-bond formed in a dimer, and *K*_n_ is the association constant for every subsequent H-bond in the chain.

For dimers and trimers, the formation of cyclic species is unfavorable due to ring strain. For larger species, however, the formation of an intramolecular H-bond leads to formation of a cyclic aggregate. At high concentrations, the cyclic aggregates open up to form the linear polymeric chains that are present in neat alcohols.^25^ Among the cyclic aggregates, the tetramer is considered to be the predominant species, but larger cyclic species are also formed. The concentration of alcohol molecules present as cyclic tetramers [c] can be expressed through the tetramerisation constant *K*_c_ (eqn (5)). We assume that the internal OH groups present in cyclic and linear aggregates have similar solvation properties and therefore the overall concentration of internal donor and acceptor groups is the sum of eqn (3) and (5).

5 [c] = 4*K*_c_[m]^4^

In order to establish the values of *K*_n_ and *K*_c_, the aggregation of 1-decanol in cyclohexane was investigated previously by following the ^1^H NMR chemical shift of the OH proton as a function alcohol concentration (see below). A good fit to a monomer–tetramer–polymer isotherm was obtained giving association constants of *α*_coop_ = 1, *K*_n_ = 2 M^–1^ and *K*_c_ = 820 M^–3^.^19^ These values agree well with other studies using various experimental techniques.^22^,^26^–^28^ For example, association constants of *α*_coop_ = 1, *K*_n_ = 0.7 M^–1^ and *K*_c_ = 660 M^–3^ were obtained for 1-octanol in *n*-octane based on the IR intensity of the OH stretching vibration.^26^

**Fig. 8:**
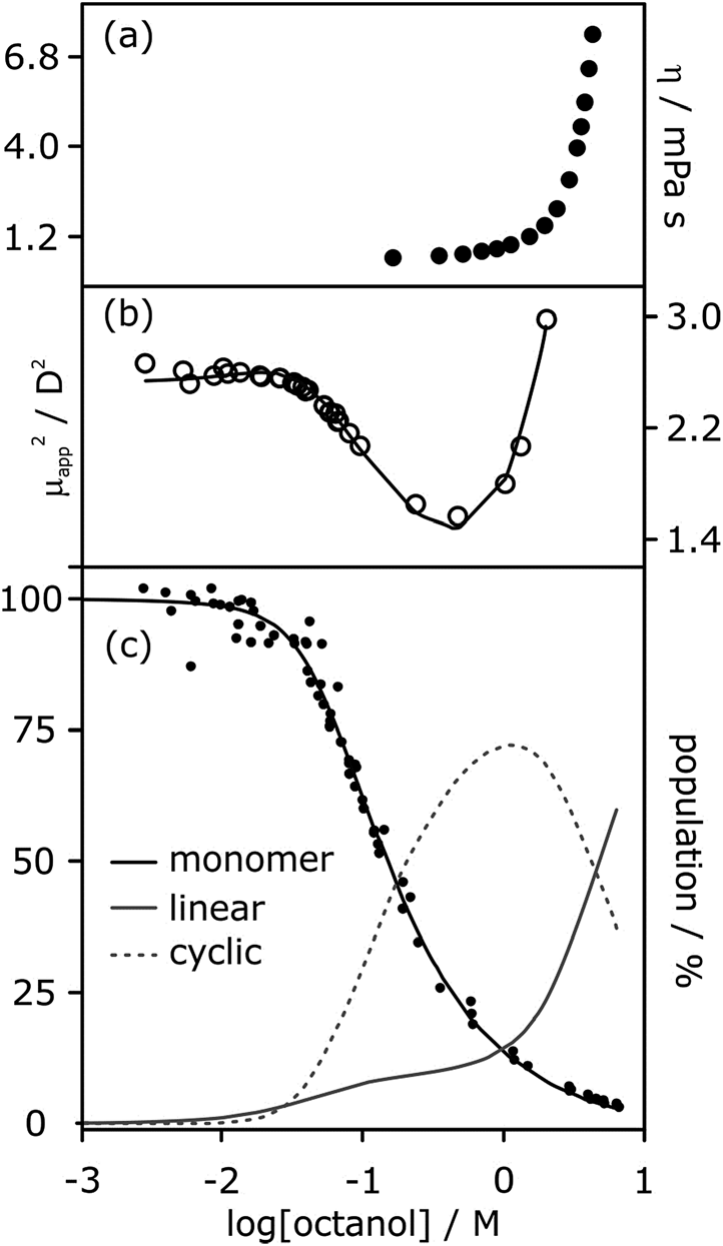
(a) Viscosity of 1-octanol in *n*-octane.^35^ (b) Apparent dipole moment of 1-octanol in cyclohexane.^29^ (c) Population of monomeric 1-octanol in *n*-octane and in *n*-decane measured by IR spectroscopy.^22^,^26^ Black lines in (b) and (c) correspond to fits of the experimental data to eqn (3)–(5) with *α*_coop_ = 9, *K*_n_ = 5 M^–1^ and *K*_c_ = 500 M^–3^. The grey lines show the populations of alcohol present as linear aggregates (solid line) and as cyclic tetramers (dashed line).

The low values of *K*_n_ determined by NMR and IR spectroscopy suggest that cyclic tetramers predominate even at high alcohol concentrations and that linear aggregates are populated to a limited extent. In contrast, viscosity data for solutions of linear alcohols in alkanes show that there are large increases in viscosity at concentrations above 1 M, indicating the presence of long polymeric aggregates rather than small cyclic species (Fig. 8a).^19^ Similar evidence comes from the apparent dipole moment of 1-octanol in cyclohexane solution determined by dielectric measurements.^29^ The dipole moment shows a minimum at a concentration of about 1 M, which is ascribed to the formation of low polarity cyclic species. At concentrations above 1 M, the dipole moment increases dramatically, indicating the formation of a different species that is more polar than the monomer, *i.e.* linear polymers (Fig. 8b).

It is possible to account for the presence of long linear polymers at high concentrations, if a slightly larger value is assigned to the linear polymerisation constant *K*_n_ (*i.e.* 3–4 rather than 1–2 M^–1^).^19^ However, this treatment would overestimate the extent of self-association at lower alcohol concentrations. A cooperative model for the polymerisation process is therefore required to reconcile the behavior at high and low concentrations, *i.e. α*_coop_ > 1 in eqn. (3) and (4). The best combined fit to the IR and dipole moment data for 1-octanol in alkanes was obtained for *α*_coop_ = 9, *K*_n_ = 5 M^–1^ and *K*_c_ = 500 M^–3^, as shown in Fig. 8. Fitting the NMR data for 1-decanol in cyclohexane using this cooperative polymerisation isotherm gives comparable values (see below). Fig. 8c shows the speciation of different aggregates based on these self-association constants. At low concentrations the major aggregate is the cyclic tetramer, but above a concentration of 1 M, there is a sharp increase in the amount of linear polymer, which is in excellent agreement with the viscosity data shown in Fig. 8a.

The value of the cooperativity factor *α*_coop_ = 9 implies that the monomer–monomer interaction is relatively weak, but once two alcohols have formed a H-bond, polarisation of the hydroxyl groups significantly increases the strength of all subsequent H-bonding interactions. Similar results have been reported for 1-hexanol in *n*-hexane,^30^,^31^ and an increase in H-bond strength due to cooperative effects has been described in diols,^32^ in carbohydrates^33^ and in phenols.^21^ An investigation of the interaction of different alcohols with pyridine N-oxide concluded that the binding constant for complexation with an alcohol dimer is approximately ten times larger than for a monomeric alcohol.^34^ These results have important implications for understanding how alcohols behave as solvents. The cooperative effects change both the speciation and the polarity of alcohol aggregates, and hence the interactions with solutes.

### Solvation properties of alcohol aggregates

**Fig. 9:**
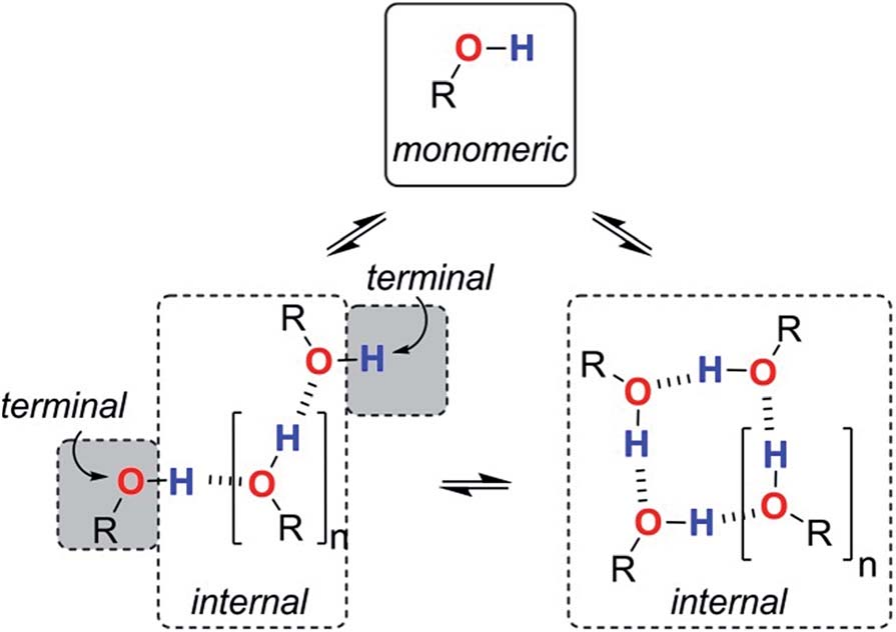
Different types of H-bond donor and acceptor site present in an alcohol solution. The internal H-bonded sites of linear and cyclic aggregates are considered to have similar properties.

We are now in a position to consider how the different aggregates of 1-octanol might affect the log *K vs.* log[S2] profiles of the molecular recognition probes discussed above. Alcohols have hydroxyl groups that could be available to interact with solutes as free monomer sites, as H-bonded internal sites in cyclic or linear aggregates, or as free terminal sites on the ends of linear chains (Fig. 9). The experiments described above suggest that compared with the monomer, the internally H-bonded sites are likely to be less polar and the terminal sites on the ends of chains are likely to be more polar. Eqn (2) should therefore be extended to account for the different solvation properties of these species by expressing the solvation of solutes by S2 as the sum of three contributions (eqn (6) and (7)).

6 *K*_A_[S2] = *K*_Am_[m] + *K*_At_[t] + *K*_Ai_[i]

7 *K*_D_[S2] = *K*_Dm_[m] + *K*_Dt_[t] + *K*_Di_[i]

where m, t and i refer to monomeric alcohol, terminal sites on the ends of linear chains, and H-bonded internal sites in cyclic or linear species respectively.

Assuming that the presence of dilute solutes does not significantly perturb the speciation of the alcohol aggregates, eqn (1)–(7) can be used to predict the log *K vs.* log[S2] profiles that we have measured in alcohol–alkane mixtures. The only missing parameters are the H-bond parameters for the terminal and internal alcohol sites that are required to calculate the solvation constants *K*_At_, *K*_Ai_, *K*_Dt_ and *K*_Di_ in eqn (6) and (7). The experiments on bifurcated H-bonding interactions with phenols **1** and **2** described above suggest that the internal OH donor sites have a H-bond donor parameter that is negligible. For the internal OH acceptor sites, on the other hand, the second lone pair of the oxygen atom remains available for interaction with solutes, and we assume that this site is unaffected by aggregation. The large cooperativity parameter found for formation of alcohol polymers (*α*_coop_ = 9) suggests that the terminal sites should have H-bond parameters that are larger than those of the monomer.

**Fig. 10:**
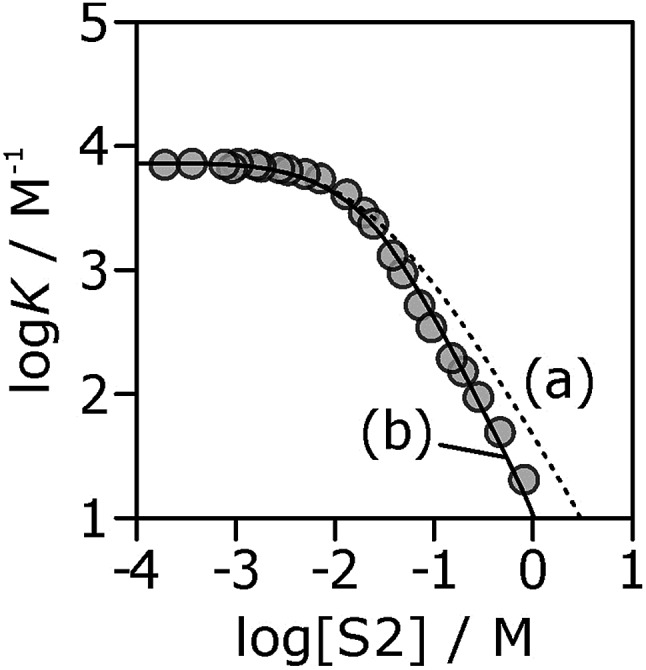
Association constants (log *K*/M^–1^) for formation of the **7·13** complex as a function of concentration of 1-octanol (S2) in *n*-octane (S1) at 298 K. The corresponding values calculated values using eqn (1)–(7) are shown for two different representations of the H-bond properties of alcohol aggregates: (a) internal OH donor sites blocked and all other sites the same as the monomer (*α*_i_ = 0, *α*_t_ = *α*_m_ = 2.7 and *β*_i_ = *β*_t_ = *β*_m_ = 5.3); (b) internal OH donor sites blocked, internal OH acceptor sites the same as the monomer, and more polar terminal sites (*α*_i_ = 0, *α*_t_ = 3.5, *α*_m_ = 2.7 and *β*_i_ = 5.3, *β*_t_ = 6.9, *β*_m_ = 5.3).

If the H-bond parameters for the terminal OH groups are set equal to the monomer values of *α*_m_ = 2.7 and *β*_m_ = 5.3, and the H-bond parameters for the internal alcohol sites are set to *α*_i_ = 0 and *β*_i_ = 5.3, log *K vs.* log[S2] profiles can be calculated using eqn (1)–(7). The calculated line labelled (a) in Fig. 10 shows the result for the **7·13** complex. This treatment underestimates the decrease in the experimentally measured log *K* values at high alcohol concentrations, *i.e.* in the concentration range where linear alcohol polymers are populated to a significant extent.

Therefore, the two H-bond parameters for terminal OH groups were allowed to vary in order to obtain the best fit to the experimental data (the calculated line labeled (b) in Fig. 10, see ESI† for data for all complexes). Using H-bond parameters for the terminal OH groups that are significantly larger than the corresponding monomer values (*α*_t_ = 3.5 ± 0.2, *β*_t_ = 6.9 ± 0.4 compared with *α*_m_ = 2.7, *β*_m_ = 5.3) provides a much better description of the experimental data. These optimised H-bond parameters can be used to calculate association constants for monomer–monomer, monomer–terminal and terminal–terminal alcohol–alcohol H-bonding interactions in *n*-octane. The ratio of the monomer–terminal and monomer–monomer association constants represents a lower limit for the cooperativity factor for the speciation of alcohol aggregates, and the ratio of the terminal–terminal and monomer–monomer association constants represents an upper limit. The result, *α*_coop_ = 4–20, is consistent with the value of 9 obtained by fitting the dilution data shown in Fig. 8.

### Polarization of H-bonded hydroxyl groups

**Fig. 11:**
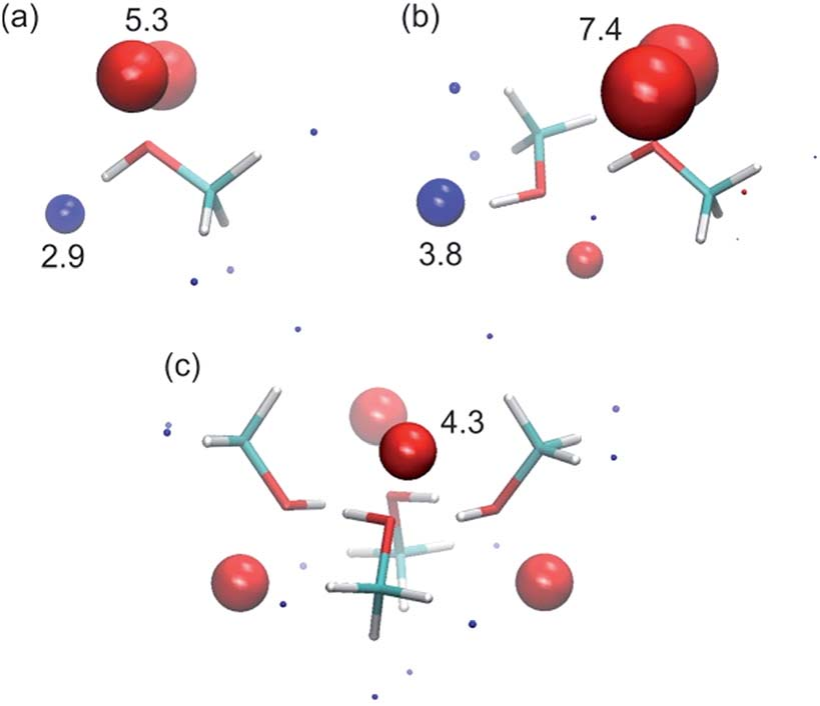
SSIP representation of (a) a methanol monomer, (b) a methanol dimer and (c) a methanol cyclic tetramer. Values of the H-bond parameters are shown for the most polar SSIPs (*α* blue sites, and *β* red sites).

To corroborate the experimental findings, *ab initio* calculations of the H-bond parameters of alcohol aggregates were carried out using the surface site interaction point (SSIP) approach.^6^Fig. 11 shows the structures of methanol aggregates optimised using DFT (B3LYP/6-31G(d)) and the SSIPs that were obtained by footprinting the molecular electrostatic potential surfaces as described previously (similar results were obtained for other alcohols, see ESI†).

The values of the H-bond parameters calculated for the most polar sites in the methanol aggregates are shown in Table 4 and agree well with the explanation used to account for the experimental data presented above. For the monomer, the calculated H-bond parameters (*α* = 2.9 and *β* = 5.3) are in good agreement with the experimental values (*α*_m_ = 2.7 and *β*_m_ = 5.3). For the H-bonded dimer of methanol, the calculated H-bond parameters for the terminal donor and acceptor sites are significantly larger (*α* = 3.8, *β* = 7.4) and in good agreement with the experimental values reported above (*α*_t_ = 3.5 and *β*_t_ = 6.9). Moreover, the proton that is involved in the methanol–methanol H-bond is buried in the dimer, resulting in a very small SSIP. The internal H-bond acceptor site, on the other hand, is predicted to still be available for H-bonding in the dimer, albeit with a lower H-bond acceptor parameter. Larger linear aggregates gave similar values (Table 4), indicating that binding of additional alcohol molecules to the chain does not strongly reinforce the polarisation, which is again consistent with the experimental behaviour.^21^ In the cyclic tetramer, all H-bond donor sites are blocked, but one internal acceptor site per alcohol is available with a slightly lower H-bond acceptor parameter.

**Table 4:** Calculated H-bond parameters for hydroxyl groups in methanol aggregates^6^

	*α*_t_	*β*_t_	*α*_i_	*β*_i_
Monomer	2.9	5.3	—	—
Linear dimer	3.8	7.4	0.4	2.9
Linear trimer	3.9	7.3	0.3	3.9
Cyclic tetramer	—	—	^*a*^	4.3

^*a*^No *α*_i_ sites predicted (see Fig. 11).

### Solvation properties of neat alcohols

**Fig. 12:**
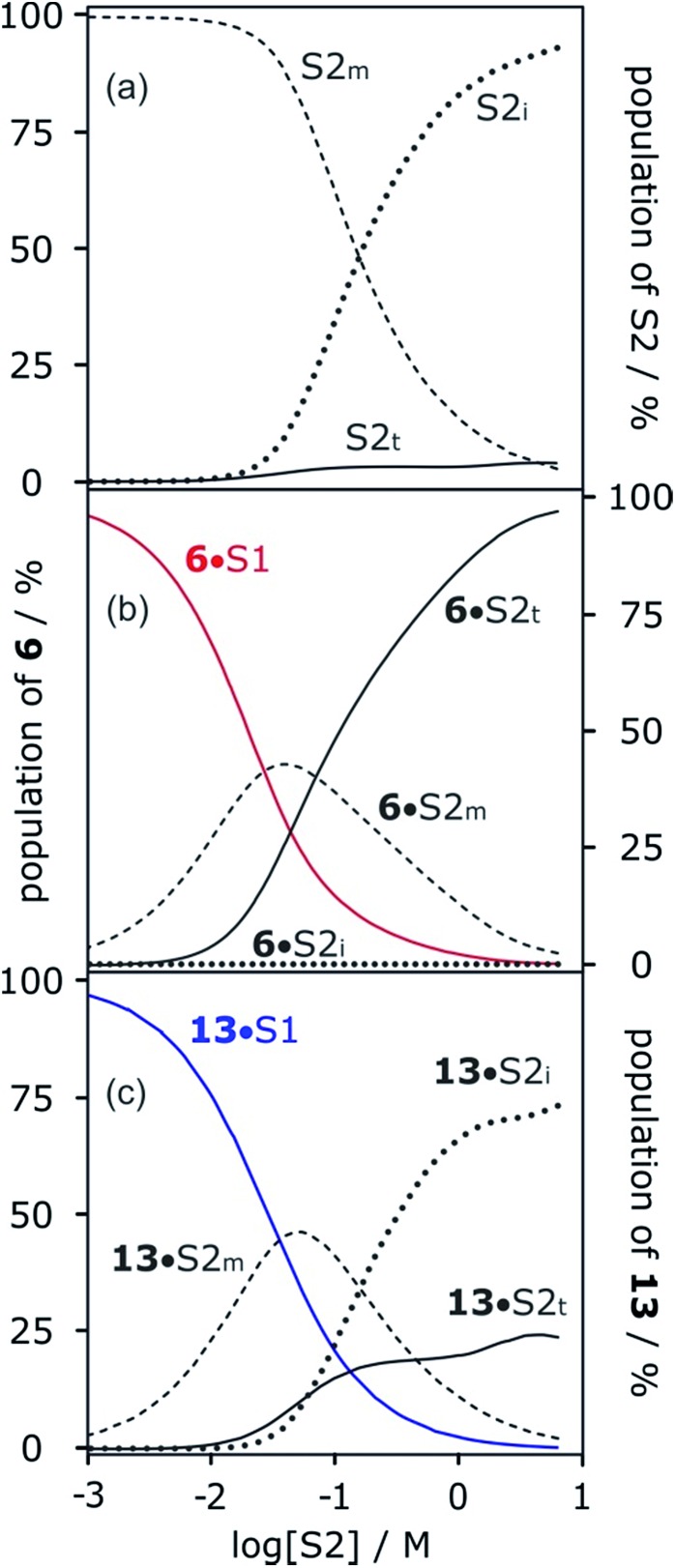
(a) Speciation of alcohol monomer (S2_m_), internal (S2_i_) and terminal (S2_t_) H-bonding sites in mixtures of 1-octanol (S2) and *n*-octane (S1). (b) Speciation of solvation states of H-bond acceptor solute **6** in mixtures of 1-octanol (S2) and *n*-octane (S1): **6**·S1 solid red line; **6**·S2_m_ dashed line; **6**·S2_i_ dotted line; **6**·S2_t_ solid black line. (c) Speciation of solvation states of H-bond donor solute **13** in mixtures of 1-octanol (S2) and *n*-octane (S1): **13**·S1 solid blue line; **13**·S2_m_ dashed line; **13**·S2_i_ dotted line; **13**·S2_t_ solid black line. Calculated using the following H-bond parameters for S2: *α*_i_ = 0, *α*_t_ = 3.5, *α*_m_ = 2.7 and *β*_i_ = 5.3, *β*_t_ = 6.9, *β*_m_ = 5.3.

The concentration-dependent speciation of different aggregation states of alcohol solvents together with the increased polarity of the terminal sites on polymeric chains determine which species are involved in solvation of solutes. Fig. 12a shows the speciation of monomer, internal and terminal H-bonding sites as a function of alcohol concentration, [S2]. The concentration of the most polar terminal sites never exceeds about 5% of the total population, but Fig. 12b shows that these species play a disproportionately important role in solvating solutes. For acceptor solutes, there are only two different solvating species involved, monomers and terminal sites. At low [S2], solvation by monomeric solvent dominates, but at higher concentrations, the more polar chain ends quickly take over. For donor solutes, the internal H-bond acceptor sites in alcohol aggregates also play a role, and these sites dominate at high values of [S2], but there is still a significant contribution from the less abundant but more polar chain ends (Fig. 12c).

**Fig. 13:**
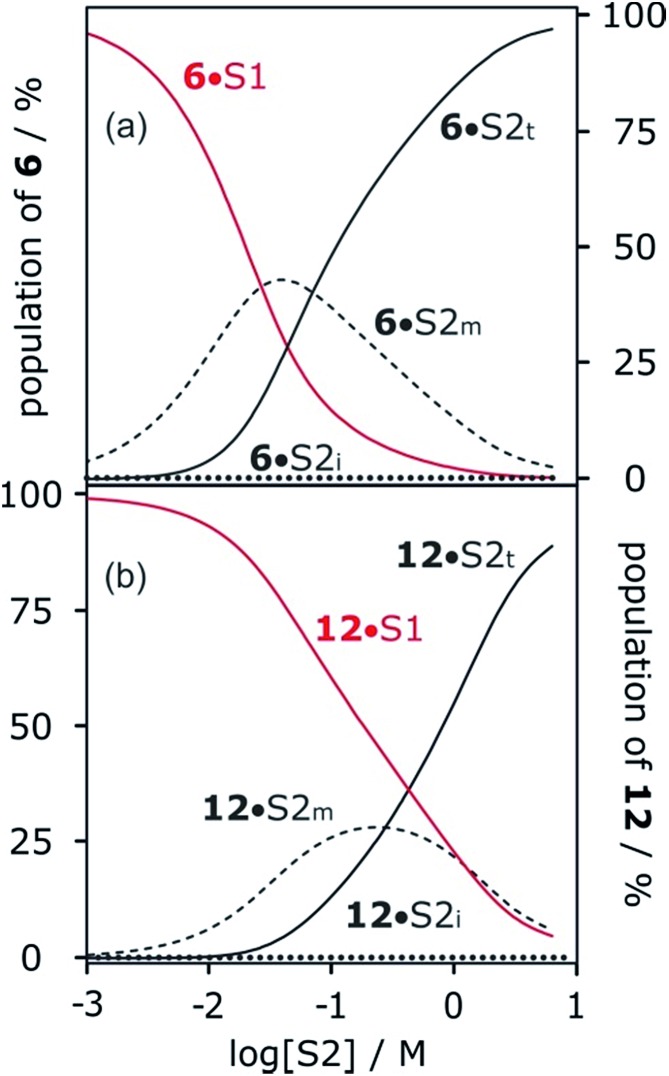
Speciation of two different H-bond acceptors (A) in mixtures of 1-octanol (S2) and *n*-octane (S1). (a) **6** (*β* = 10.7). (b) **12** (*β* = 7.8). A·S1 solid red lines; A·S2_m_ dashed lines; A·S2_i_ dotted lines; A·S2_t_ solid black lines. Calculated using the following H-bond parameters for S2: *α*_i_ = 0, *α*_t_ = 3.5, *α*_m_ = 2.7 and *β*_i_ = 5.3, *β*_t_ = 6.9, *β*_m_ = 5.3.

Fig. 13 shows the speciation of solvation states for two different H-bond acceptors as a function of alcohol concentration. For the more polar solute **6** (Fig. 13a), the H-bonding interactions with the solvent are stronger, so preferential solvation by the alcohol starts at lower values of [S2], where the solvent monomer is the major species. For the less polar solute **12** (Fig. 13b), preferential solvation by the alcohol occurs at a higher value of [S2], where the formation of alcohol aggregates competes for interactions with the solute. The result is that preferential solvation of less polar solutes shows a much weaker dependence on [S2] than for more polar solutes (compare the slopes of the populations of A·S1 for **6** and **12** in Fig. 13). The difference in the nature of the alcohol species responsible for preferential solvation of solutes is the origin of differences in slope observed in the log *K vs.* log[S2] profiles that are reported in these experiments show that the slope of approximately –2 that was previously reported for the high [S2] regime of the log *K vs.* log[S2] profile for the **6·13** complex is not a general property of the alcohol solvent. The value of the slope also depends on the nature of the solutes. For less polar solutes, the slope is –1, which is the same as the value observed in mixtures of alkanes and polar solvents that do not self-associate. These results indicate that there is an interplay between solvent self-association and solute polarity that leads to qualitative differences between the nature of the solvation shells in these systems.

More polar solutes have more negative slopes because they are solvated predominantly by monomers. It may seem counterintuitive that the less polar monomeric alcohols solvate solutes more strongly than the polar chain ends of aggregated alcohols. The reason is that in the concentration range where preferential solvation occurs ([S2] < 100 mM), the concentration of chain ends is much lower than the concentration of monomers (Fig. 12a).

Fig. 12 shows that at high alcohol concentrations both of the solutes are entirely solvated by linear aggregates that are considerably more polar than the monomer. Thus, the H-bond parameters of the aggregates can be used to rationalise the solvation properties of neat alcohols. Previously, we reported the association constants for a complex formed between phosphine oxide **6** and perfluoro-*tert*-butyl alcohol in thirteen different solvents. Eqn (1) accurately predicted the experimental values for all solvents, with the exception of 1-decanol. 1-Decanol was the only alcohol solvent in which measurements could be made, and it was found to be much more polar than expected based on the H-bond parameters of monomeric alcohols (log *K*_expt_ = –0.7 ± 1 compared with log *K*_calc_ = 0.8). However, using the values of *α*_t_ = 3.5 and *β*_t_ = 6.9 as *α*_S_ and *β*_S_ in eqn (1) gives a value of log *K* = –0.1, which is consistent with the experimental value.

### Solvation properties of different alcohols

**Fig. 14:**
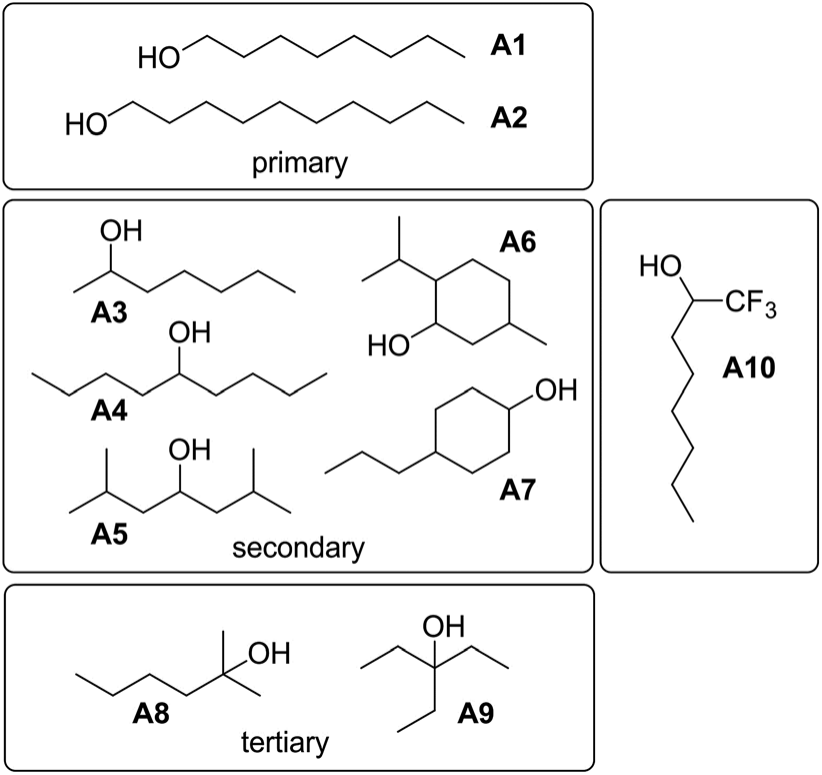
Alcohols **A1–A10** used as S2 in mixed solvent titrations.

To investigate the effects of varying the nature of the alcohol solvent, the experiments on the **6·13** complex were repeated using a range of different alcohols as S2 in mixed solvent titrations with *n*-octane (Fig. 14). Two primary alcohols, **A1** and **A2**, five secondary alcohols, **A3–A7** (two of which are cyclohexanols), and two tertiary alcohols, **A8** and **A9**, were used to vary the steric demand around the hydroxyl H-bonding sites. To assess electronic effects, a fluorinated alcohol, trifluoro-2-octanol **A10** was also included in the study.

**Fig. 15:**
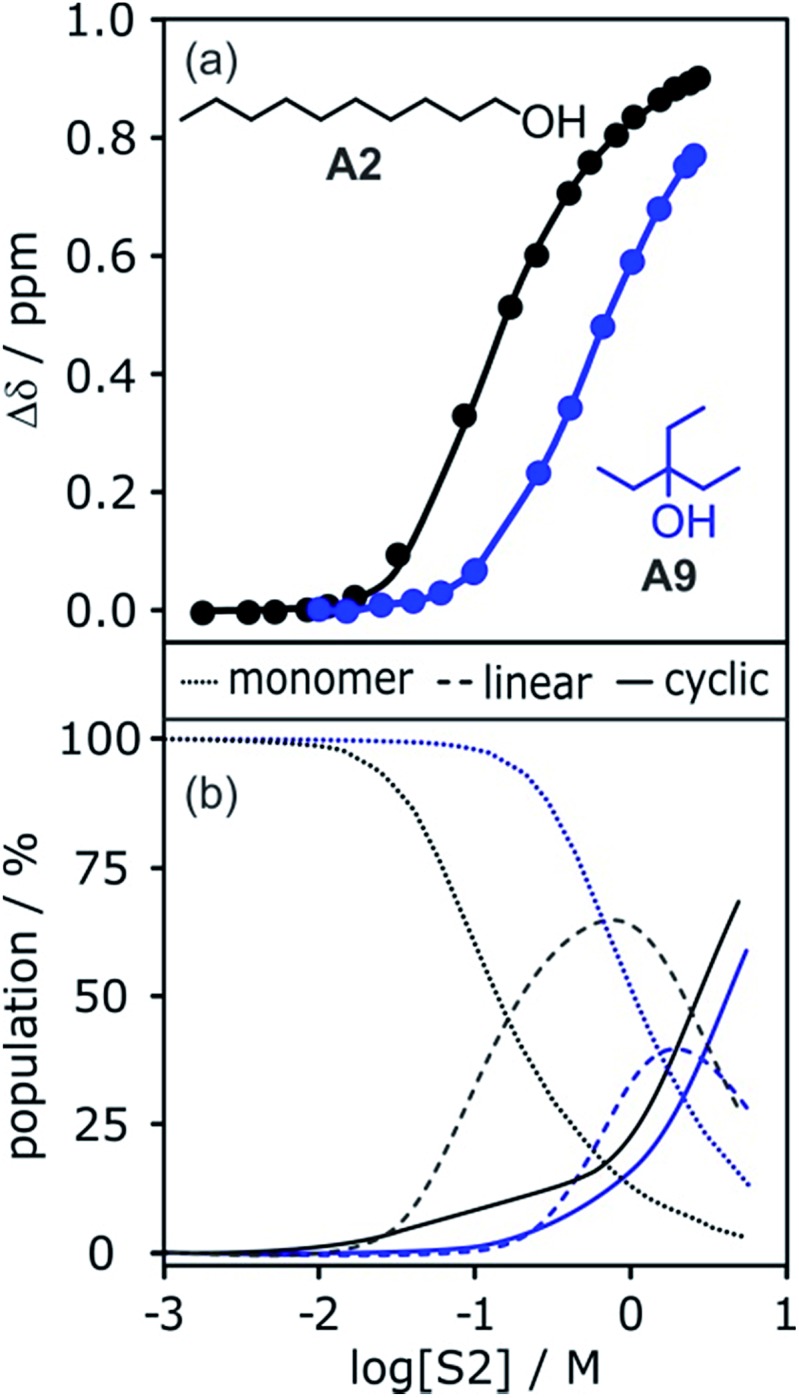
(a) ^1^H NMR dilution data for 1-decanol **A2** in d_12_-cyclohexane (black)^19^ and for 3-ethyl-3-pentanol **A9** in *n*-octane (blue). [S2] is the concentration of the alcohol. Points represent the change in the ^1^H NMR chemical shift of the signal due to the OH group as a function of alcohol concentration. Solid lines are fits of the experimental data with *α*_coop_ = 13, *K*_n_ = 7 M^–1^ and *K*_c_ = 590 M^–3^ for **A2** and *α*_coop_ = 6, *K*_n_ = 2 M^–1^ and *K*_c_ = 7 M^–3^ for **A9**. (b) The corresponding speciation profiles are shown for comparison (dotted line, monomers; solid line, linear polymers; dashed line, cyclic tetramers).

The increased steric demand of the substituents in secondary and tertiary alcohols might be expected to impact on the stability of aggregated species. IR spectroscopy,^36^,^37^ heat capacity measurements^27^,^38^–^41^ and dielectric studies^42^,^43^ all suggest that there is less self-association in branched alcohols. ^1^H NMR dilution data for the tertiary alcohol 3-ethyl-3-pentanol **A9** is compared with the primary alcohol 1-decanol **A2** in Fig. 15.

The data for **A9** can be fitted to an isotherm that takes into account cooperative formation of linear polymers as well as cyclic tetramers, yielding values of *α*_coop_ = 6, *K*_n_ = 2 M^–1^ and *K*_c_ = 7 M^–3^. The major difference compared to the linear alcohols **A1** and **A2** is that the formation of the cyclic tetramer is less favourable for the tertiary alcohol **A9**. Nevertheless, the cyclic tetramer is present in reasonable amounts at high alcohol concentrations and is the motif found in the X-ray crystal structure of **A9**.^44^ The cooperativity factor *α*_coop_ is also slightly lower for the more sterically hindered alcohol in agreement with the literature.^34^ The speciation profiles in Fig. 15 show that at concentrations where approximately half of **A2** is aggregated (log[alcohol] ≈ –1), **A9** is still mostly monomer. Thus the speciation of different alcohol aggregates can vary significantly with the structure of the alcohol. These conclusions are supported by IR spectroscopic data^37^ and dielectric measurements^45^ (see ESI†).

**Fig. 16:**
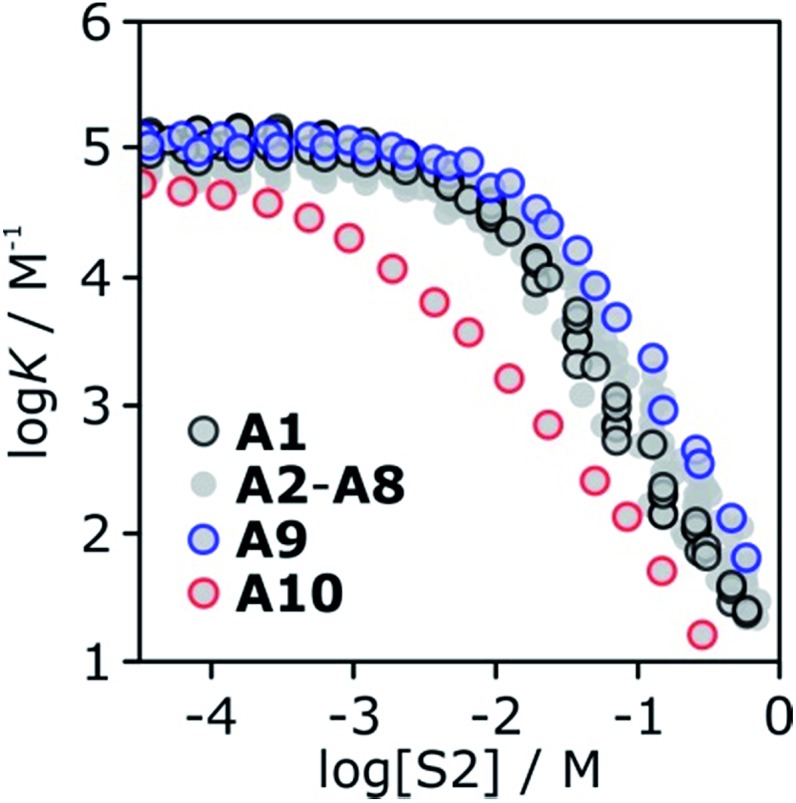
Association constants (log *K*/M^–1^) for formation of the **6·13** complex as a function of alcohol concentration (S2) in *n*-octane (S1) at 298 K. Data highlighted are 1-octanol **A1** (black), 3-ethyl-3-pentanol **A9** (blue) and trifluoromethyl-2-octanol **A10** (red).

The association constant for formation of the 1 : 1 complex between tri-*n*-butyl phosphine oxide **6** and 4-phenyl azophenol **13** was measured in binary mixtures of *n*-octane (S1) and each of the ten alcohols (S2) using automated UV-vis titrations. The results are shown in Fig. 16. The relationship between log *K* and log[S2] is the same as that illustrated in Fig. 2. At low concentrations of S2, the value of log *K* is constant, and once sufficient S2 has been added, log *K* decreases with increasing [S2]. The log *K vs.* log[S2] profiles are very similar for alcohols **A1–A9**. Interestingly, the substantial difference in the speciation of alcohol aggregates shown in Fig. 15 does not translate into a substantial difference in the log *K vs.* log[S2] profiles in Fig. 16. The biggest variation is observed between primary alcohols and tertiary alcohols, as highlighted by the black and blue data points in Fig. 16. For **A1** and **A9**, there is a difference of approximately one in the value of log *K* at [S2] = 100 mM (these values were corroborated by manual titrations for this solvent mixture). At this concentration, **A9** is mainly monomeric, whereas **A1** is 50% aggregated. Compared with **A9**, in **A1** there are fewer monomeric H-bond donors available to solvate the solute, but there are more of the very polar polymer chain ends. These two effects more or less cancel, so that the properties of the two solvents are similar.

Compared to the alkyl alcohols, the fluorinated alcohol **A10** is a better H-bond donor and a weaker acceptor and thus the solvation behavior is very different (Fig. 16, red data points). Due to the enhanced H-bond donor properties of **A10**, the onset of the decrease in log *K* is shifted to lower S2 concentrations. In this region of the log *K vs.* log[S2] profile, the slope is less steep than for the other alcohols, because the fluorinated alcohol only solvates the acceptor solute, and not the donor. At very high concentrations of **A10**, both the donor and acceptor solutes are solvated by S2, and the slope is similar to that observed for the other alcohols.

The experiments in Fig. 16 show that in the absence of strong electronic effects from substituents, the solvation properties of different alcohols are broadly similar. In contrast, the shape of the log *K vs.* log[S2] profiles differ significantly for different solutes (see Fig. 5 and 6). Specifically, the slope in the regime where alcohol solvation competes with complexation depends on the H-bond properties of the solutes: more polar solutes lead to a stronger dependence of the association constant on alcohol concentration.

## Conclusions

Alcohols are a unique class of solvents. The bulk liquids are significantly more polar than expected based on the properties of monomeric alcohol molecules in dilute solution. At high concentrations alcohols self-associate to form a variety of different aggregates, and these aggregates are responsible for enhanced interactions with solutes. The experiments described here show that these properties are unlikely to be due to bifurcated H-bonding interactions with OH groups that are already engaged in H-bonding in aggregates. Specifically, the strength of a three-centre bifurcated H-bond formed with a hydroxyl H-bond donor that is involved in an intramolecular H-bond is reduced by about three orders of magnitude compared with a two-centre H-bond.

Mixed solvent experiments using a range of solutes of different polarity in a range of different alcohols have allowed us to dissect the complex equilibria present in alcohol solutions. Molecular recognition probes were used to quantify the solvation properties of mixtures of alcohols and alkanes covering a wide range of different solvent compositions. The results indicate that the H-bonding properties of hydroxyl groups present in different types of alcohol aggregates differ significantly. The enhanced solvation properties of alcohol aggregates are due to an increase in the polarity of the terminal hydroxyl groups on the ends of linear polymeric chains. Formation of an alcohol–alcohol H-bond polarises the hydroxyl groups, increasing the H-bond donor parameter *α* from 2.7 to 3.5 and increasing the H-bond acceptor parameter *β* from 5.3 to 6.9. The increase in polarity takes place on formation of the first hydroxyl–hydroxyl H-bond in a chain and does not increase further in longer H-bonded chains. These observations are supported by *ab initio* calculation of the H-bond parameters for alcohol aggregates and account for the cooperative binding isotherm found for alcohol self-association. This phenomenon appears to be a general property of alcohols and is not affected by substituents.

## Experimental section

### Automated UV-vis titrations

Association constants were determined using a BMG Labtech Fluostar Optima plate reader equipped with a UV-vis detector and two internal injection pumps. In a typical experiment, a 96-well Hellma quartz microplate is partially loaded with equal aliquots of a solution of the respective H-bond donor in octane, followed by different volumes of H-bond acceptor solution in octane. Pure octane is then added to reach the same volume in each well. At this point the concentration of donor in each well is 100 μM, and the concentration of acceptor ranges from 0 to 407 mM over 20 wells. By using the internal injection pumps, several aliquots of alcohol solution in octane with different alcohol concentrations are successively added to each of the wells. The UV-vis spectrum of each well is recorded before and after addition of alcohol to give multiple titration datasets. Each dataset is fitted to 1 : 1 binding isotherms to obtain 27 association constants, associated to alcohol concentration between 97 μM to 806 mM.

### Manual UV-vis and NMR titrations

UV-vis spectra were recorded using an Agilent Cary 60 UV-vis spectrophotometer. NMR spectra were recorded using a Bruker Avance III HD 500 MHz Smart Probe spectrometer. In a typical experiment, a quartz cuvette or a NMR tube was filled with 1–2 mL of a 100 μM solution of the respective host in octane and aliquots of a guest solution were successively added. The host solution was used to prepare the solution of guest (35 mM), so that the concentration of the host remained constant throughout the titration. A spectrum was recorded after each addition of guest and the changes in the UV-vis or NMR signals of the host were fitted to a 1 : 1 binding isotherm using a purpose written software to obtain the equilibrium constant, *K*, for the host–guest complex.

## Conflicts of interest

There are no conflicts to declare.

## Supplementary Material

Supplementary information
