# Supplementary material for: Polarisation effects on the solvation properties of alcohols
Source: Chem Sci. 2017 Dec 6;9(1):88–99. doi: 10.1039/c7sc04890d (PMC5875020; doi:10.1039/c7sc04890d)
Supplement: Supplementary file 1 [file SC-009-C7SC04890D-s001.pdf]

Electronic Supplementary Material (ESI) for Chemical Science.
This journal is © The Royal Society of Chemistry 2017

SUPPORTING INFORMATION

Polarisation Effects on the Solvation Properties of Alcohols

Stefan Henkel, Maria Cristina Misuraca, Pavle Troselj, Jonathan Davidson, Christopher A. Hunter*

Department of Chemistry, University of Cambridge, Lensfield Road, Cambridge CB2 1EW, UK

Contents

BINDING ISOTHERMS

EXPERIMENTAL AND PREDICTED ASSOCIATION CONSTANTS

SPECIATION OF ALCOHOL AGGREGATES

H-BOND PARAMETERS FOR ALCOHOL MONOMERS AND DIMERS

OPTIMIZED GEOMETRIES

BINDING ISOTHERMS for the formation of 1:1 complexes of phenols **1**-**5** with phosphine oxide **6** in *n*-octane at 298 K.

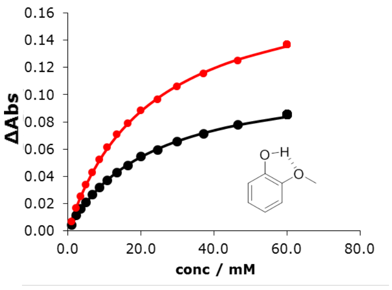

Figure S1. Fit of the experimental shifts of the UV-vis signals of 2-methoxyphenol upon titration with tri-*n*-butyl phosphine oxide.

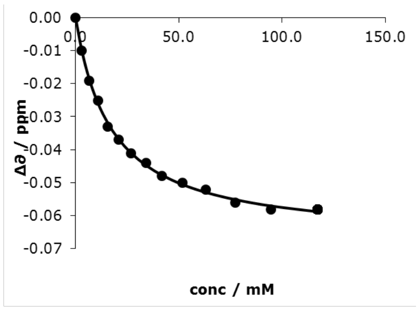

Figure S2. Fit of the experimental shifts of the ^{1}H NMR signals of the methyl protons of 2-methoxyphenol upon titration with tri-*n*-butyl phosphine oxide.

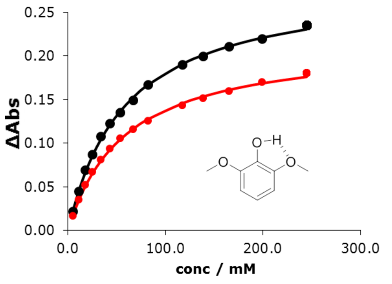

Figure S3. Fit of the experimental shifts of the UV-vis signals of 2,6-dimethoxyphenol upon titration with tri-*n*-butyl phosphine oxide.

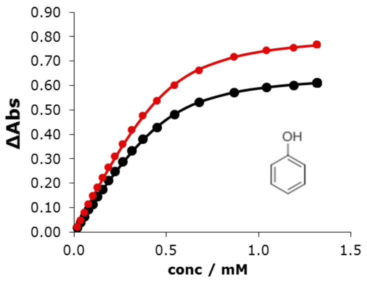

Figure S4. Fit of the experimental shifts of the UV-vis signals of phenol upon titration with tri-*n*-butyl phosphine oxide.

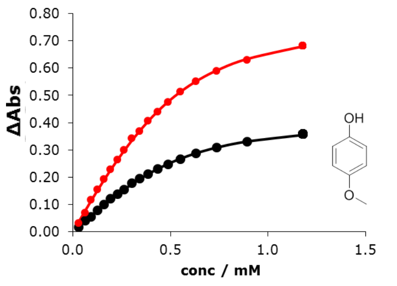

Figure S5. Fit of the experimental shifts of the UV-vis signals of 4-methoxyphenol upon titration with tri-*n*-butyl phosphine oxide.

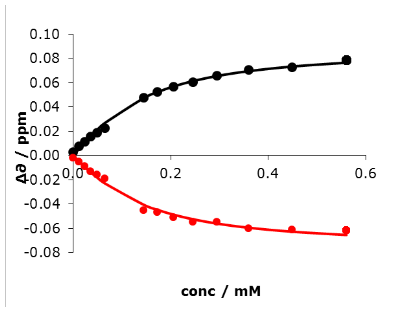

Figure S6. Fit of the experimental shifts of the ^{1}H NMR signals of the aromatic protons of 4-methoxyphenol upon titration with tri-*n*-butyl phosphine oxide.

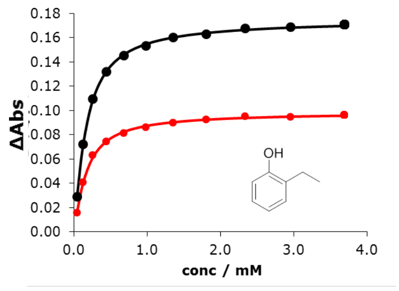

Figure S7. Fit of the experimental shifts of the UV-vis signals of 2-ethylphenol upon titration with tri-*n*-butyl phosphine oxide.

EXPERIMENTAL AND PREDICTED ASSOCIATION CONSTANTS of donors **12**-**14** and acceptors **5**-**11** the as a function of concentration of 1-octanol (S2) in *n*-octane (S1) at 298 K.

	H- bond donors		
H- bond acceptors	**13**	**14**	**15**
**6**	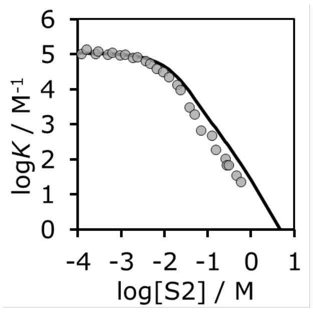	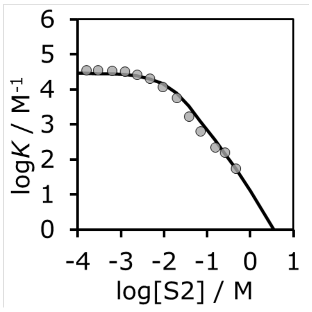	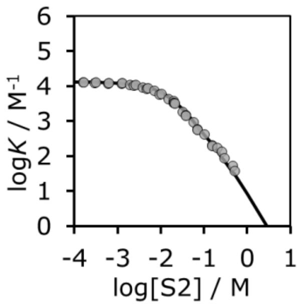
**7**	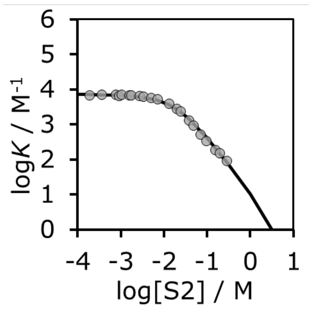	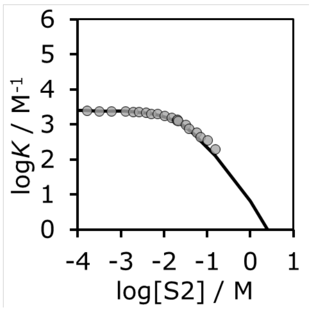	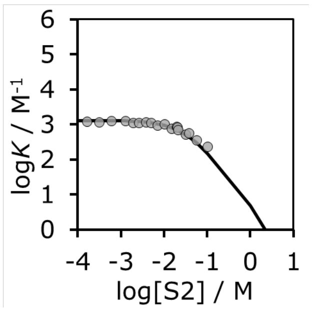
**8**	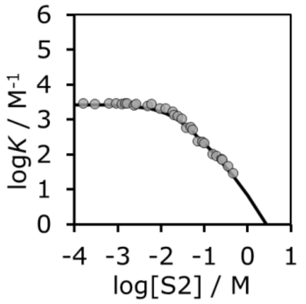	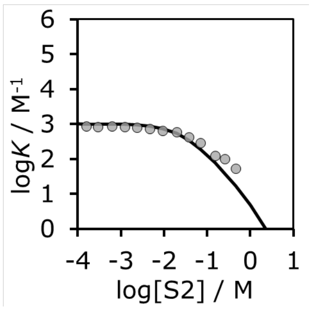	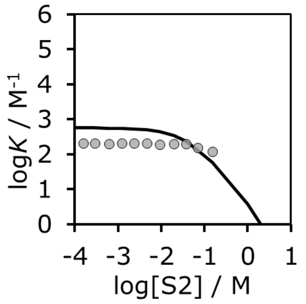
**9**	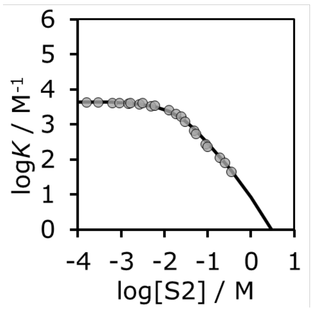	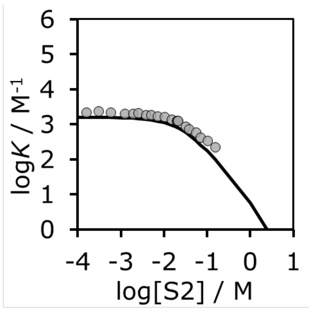	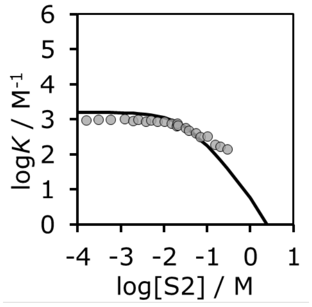
**10**	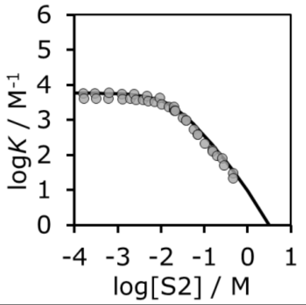	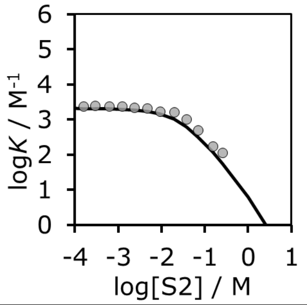	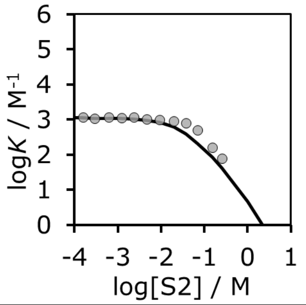

	H- bond donors		
	**13**	**14**	**15**
H- bond acceptors **11**	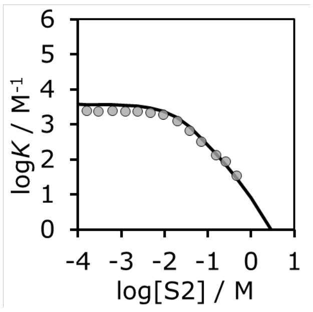	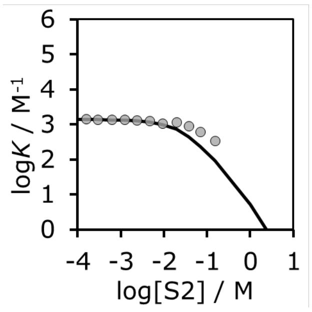	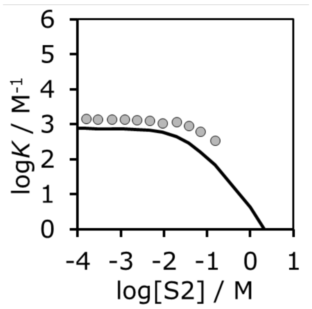
**12**	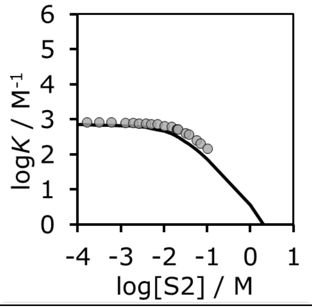	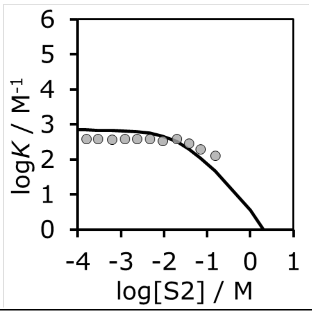	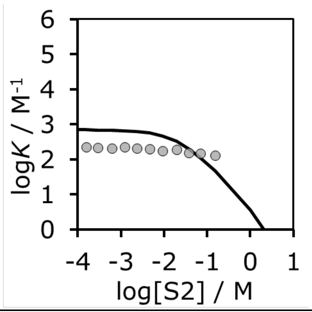

SPECIATION OF ALCOHOL AGGREGATES for 1-octanol **A1** and 3-ethyl-3-pentanol **A9** in tetrachloromethane based on literature data.

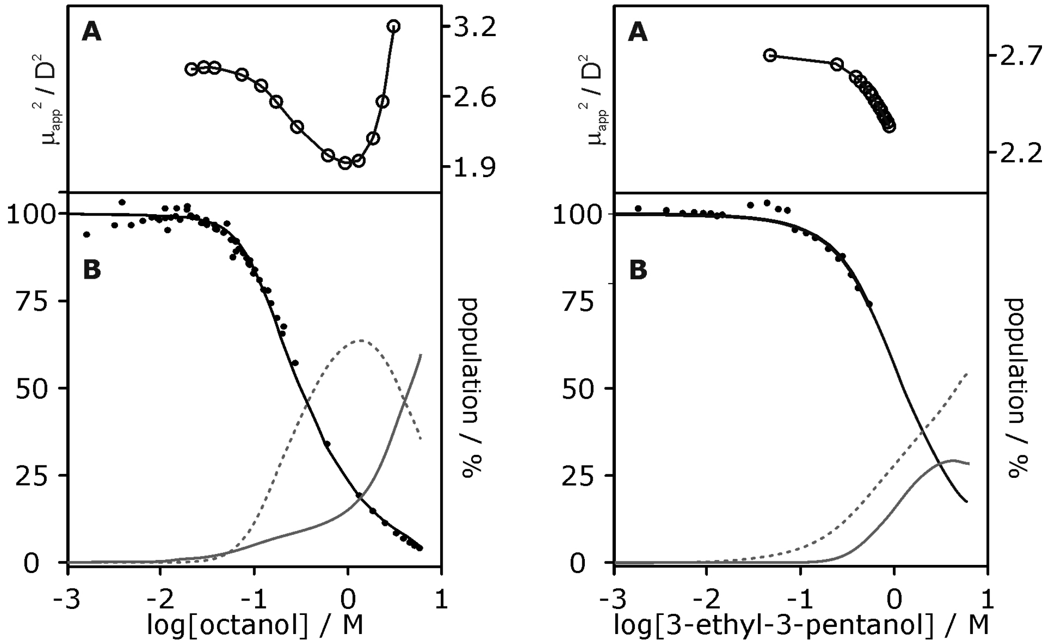

Figure S8. *Left:* **A**: Apparent dipole moment of 1-octanol in tetrachloromethane.[1] **B**: Population of monomeric 1-octanol in tetrachloromethane measured by IR spectroscopy.[2-4] *Right:* **A**: Apparent dipole moment of 3-ethyl-3-pentanol in tetrachloromethane.[5] **B**: Population of monomeric 3-ethyl-3-pentanol in tetrachloromethane measured by IR spectroscopy.[4] Black lines correspond to fits of the experimental data with K_n = 3 M^{-1}, α_c = 13 and K_4 = 75 M^{-3} for 1-octanol and K_n = 0.7 M^{-1}, α_c = 3.4 and K_4 = 0.4 M^{-3} for 3-ethyl-3-pentanol. The grey lines show the populations of linear aggregates (solid line) and cyclic tetramer (dashed line).

References

[1] Campbell, C.; Brink, G.; Glasser, L., *J. Phys. Chem.* **1975**, *79*, 660.

[2] Fletcher, A. N., *J. Phys. Chem.* **1969**, *73*, 2217.

[3] Brink, G.; Drought, Z.; Glasser, L., *S. Afr. J. Chem.* **1986**, *39*, 163-168.

[4] Nodland, E., *Appl. Spectrosc.* **2000**, *54*, 1339.

[5] Kunst, M.; Vanduijn, D.; Bordewijk, P., *Ber. Bunsen Phys. Chem.* **1976**, *80*, 839.

H-BOND PARAMETERS FOR ALCOHOL MONOMERS AND DIMERS calculated from the molecular electrostatic potential.

Table S1. H-bond parameters for alcohols **A1**-**A10** calculated for the surface site interaction points at the B3LYP/6-31G(d) level of theory.

		Monomer		Dimer	
		α	β	α	β
1-Octanol	**A1**	2.8	5.3	3.5	7.4
1-Decanol	**A2**	2.8	5.3	3.5	7.4
2-Heptanol	**A3**	2.7	5.3	3.3	6.9
5-Nonanol	**A4**	2.7	5.1	3.3	5.8
2,4-Dimethyl-4-heptanol	**A5**	2.7	4.9	3.2	5.3
Menthol	**A6**	2.7	5.1	3.5	5.9
4-Penty-cyclohexanol	**A7**	2.7	5.3	3.3	6.5
2-Methyl-2-hexanol	**A8**	2.6	5.3	3.2	6.4
3-Ethyl-3-pentanol	**A9**	2.7	5.1	3.3	6.7
1,1,1-Trifluoro-2-octanol	**A10**	3.2	3.5	4.0	5.7

OPTIMIZED GEOMETRIES of monomer and dimers of alcohols **A1**-**A10** and methanol. Geometry optimizations, frequency calculations and calculations of the electrostatic potential were carried out using the B3LYP function and the 6-31G* basis set as implemented in Gaussian 03.

(Gaussian 03, Revision E.01; Frisch, M. J. T., G. W.; Schlegel, H. B.; Scuseria, G. E.; Robb, M. A.; Cheeseman, J. R.; Montgomery, Jr., J. A.; Vreven, T.; Kudin, K. N.; Burant, J. C.; Millam, J. M.; Iyengar, S. S.; Tomasi, J.; Barone, V.; Mennucci, B.; Cossi, M.; Scalmani, G.; Rega, N.; Petersson, G. A.; Nakatsuji, H.; Hada, M.; Ehara, M.; Toyota, K.; Fukuda, R.; Hasegawa, J.; Ishida, M.; Nakajima, T.; Honda, Y.; Kitao, O.; Nakai, H.; Klene, M.; Li, X.; Knox, J. E.; Hratchian, H. P.; Cross, J. B.; Bakken, V.; Adamo, C.; Jaramillo, J.; Gomperts, R.; Stratmann, R. E.; Yazyev, O.; Austin, A. J.; Cammi, R.; Pomelli, C.; Ochterski, J. W.; Ayala, P. Y.; Morokuma, K.; Voth, G. A.; Salvador, P.; Dannenberg, J. J.; Zakrzewski, V. G.; Dapprich, S.; Daniels, A. D.; Strain, M. C.; Farkas, O.; Malick, D. K.; Rabuck, A. D.; Raghavachari, K.; Foresman, J. B.; Ortiz, J. V.; Cui, Q.; Baboul, A. G.; Clifford, S.; Cioslowski, J.; Stefanov, B. B.; Liu, G.; Liashenko, A.; Piskorz, P.; Komaromi, I.; Martin, R. L.; Fox, D. J.; Keith, T.; Al-Laham, M. A.; Peng, C. Y.; Nanayakkara, A.; Challacombe, M.; Gill, P. M. W.; Johnson, B.; Chen, W.; Wong, M. W.; Gonzalez, C.; Pople, J. A. Gaussian, Inc.: Pittsburgh PA, 2003.)

Methanol

Monomer				Dimer			
C	-0.511	0.316	0.000	H	0.372	2.995	-0.092
H	-0.154	-0.693	0.000	O	1.123	2.648	-0.612
H	-0.154	0.820	-0.874	H	-1.817	3.861	0.944
H	-1.581	0.316	0.000	O	-1.316	3.061	0.724
O	-0.034	0.990	1.168	C	2.000	1.971	0.292
H	-0.352	1.896	1.167	H	2.835	1.578	-0.248
				H	2.347	2.659	1.035
				H	1.473	1.169	0.767
				C	-2.123	2.242	-0.127
				H	-2.360	2.782	-1.020
				H	-1.585	1.352	-0.380
				H	-3.026	1.980	0.382

Trimer				Cyclic tetramer			
H	0.372	2.995	-0.092	C	0.121	2.783	1.138
O	1.123	2.648	-0.612	H	-0.764	3.418	1.183
C	2.000	1.971	0.292	H	0.995	3.436	1.059
H	2.835	1.578	-0.248	H	0.190	2.235	2.083
H	2.347	2.659	1.035	O	-0.002	1.928	0.024
H	1.473	1.169	0.767	H	0.777	1.322	-0.008
H	-1.826	3.874	0.889	C	-2.791	0.123	-1.135
O	-1.340	3.038	0.744	H	-3.425	-0.762	-1.181
H	-2.745	6.240	1.302	H	-2.250	0.199	-2.084
O	-2.393	5.642	0.625	H	-3.445	0.996	-1.046
C	-2.146	2.209	-0.097	O	-1.928	-0.005	-0.028
H	-1.640	1.284	-0.276	H	-1.322	0.773	0.005
H	-3.083	2.017	0.383	C	2.788	-0.122	-1.136
H	-2.317	2.707	-1.029	H	3.423	0.763	-1.182
C	-1.256	6.264	0.020	H	2.242	-0.192	-2.082
H	-0.512	6.448	0.767	H	3.442	-0.995	-1.056
H	-0.854	5.617	-0.732	O	1.932	0.002	-0.024
H	-1.551	7.190	-0.426	H	1.326	-0.776	0.008
				C	-0.125	-2.780	1.140
				H	0.760	-3.413	1.197
				H	-0.997	-3.435	1.057
				H	-0.203	-2.227	2.081
				O	0.006	-1.931	0.022
				H	-0.772	-1.325	-0.018

1-Octanol **A1**							
Monomer				Dimer			
C	-3.764	0.450	0.000	H	0.372741	2.996967	-0.091911
H	-3.796	1.104	0.913	O	1.122719	2.648454	-0.611509
H	-3.796	1.104	-0.913	C	1.992181	1.976722	0.284767
C	-2.512	-0.409	0.000	C	3.123377	1.342321	-0.516714
C	-1.272	0.458	0.000	H	1.457628	1.194302	0.851784
H	-2.521	-1.074	-0.902	H	2.416195	2.673288	1.030060
H	-2.521	-1.074	0.902	H	-1.817467	3.860282	0.944951
H	-1.277	1.125	-0.902	O	-1.315777	3.060691	0.723950
C	-0.016	-0.387	0.000	C	-2.125856	2.238958	-0.130491
H	-1.277	1.124	0.903	H	-1.442903	1.471132	-0.504812
H	-0.012	-1.053	-0.903	H	-2.473665	2.816095	-1.000691
H	-0.011	-1.053	0.902	C	-3.306019	1.614610	0.609439
C	1.226	0.477	0.000	H	2.682285	0.667406	-1.262828
H	1.221	1.144	-0.903	C	4.127960	0.580246	0.355024
C	2.482	-0.367	0.000	H	3.635526	2.134396	-1.079552
H	1.221	1.144	0.902	H	3.601082	-0.202201	0.921866
H	2.488	-1.034	-0.903	H	4.554049	1.262389	1.105982
H	2.488	-1.034	0.902	C	5.268597	-0.061339	-0.445994
C	3.727	0.496	0.000	H	4.844284	-0.740527	-1.200216
H	3.719	1.162	-0.903	C	6.269353	-0.831805	0.424527
C	4.980	-0.341	0.000	H	5.801224	0.719884	-1.008230
H	3.718	1.163	0.902	H	5.734908	-1.612726	0.986166
H	5.018	-0.995	-0.904	H	6.691811	-0.152321	1.179898
H	5.017	-0.995	0.905	C	7.412074	-1.474556	-0.372026
H	5.884	0.314	0.000	H	6.991363	-2.153443	-1.128948
O	-4.875	-0.433	0.000	C	8.409202	-2.247405	0.500700
H	-5.665	0.119	-0.001	H	7.949745	-0.694430	-0.931639
				H	7.872130	-3.027995	1.058403
				H	8.828746	-1.569122	1.257517
				C	9.549216	-2.885045	-0.300107
				H	9.164026	-3.594807	-1.042700
				H	10.242132	-3.429261	0.351998
				H	10.127732	-2.125155	-0.840007
				C	-4.133949	0.675664	-0.278322
				H	-3.952939	2.414834	1.001004
				H	-2.921225	1.069993	1.481479
				H	-4.499927	1.228086	-1.156382
				C	-5.324653	0.041525	0.452647
				H	-3.482852	-0.119601	-0.669488
				H	-5.978418	0.836813	0.840373
				H	-4.958626	-0.506694	1.333072
				C	-6.147343	-0.906284	-0.429605
				H	-6.511162	-0.357924	-1.311284
				C	-7.338862	-1.541238	0.298691
				H	-5.492407	-1.701153	-0.816223
				H	-7.995835	-0.746855	0.683560
				H	-6.975961	-2.088157	1.181749
				C	-8.159680	-2.492240	-0.581962
				H	-8.521056	-1.946193	-1.465031
				C	-9.349014	-3.120328	0.151817
				H	-7.503885	-3.287325	-0.964444
				H	-10.043190	-2.351428	0.512958
				H	-9.016693	-3.699634	1.022118
				H	-9.911922	-3.795868	-0.502384

1-Decanol **A2**							
Monomer				Dimer			
C	-3.764	0.450	0.000	H	0.372067	3.023060	-0.088831
H	-3.796	1.104	0.913	O	1.122230	2.674763	-0.608316
H	-3.796	1.104	-0.913	C	1.990854	2.001517	0.287648
C	-2.512	-0.409	0.000	C	3.119731	1.363830	-0.514504
C	-1.272	0.458	0.000	H	1.454905	1.220753	0.855604
H	-2.521	-1.074	-0.902	H	2.417212	2.697526	1.032128
H	-2.521	-1.074	0.902	H	-1.821145	3.882311	0.945853
H	-1.277	1.125	-0.902	O	-1.317317	3.083938	0.725337
C	-0.016	-0.387	0.000	C	-2.124822	2.259957	-0.129421
H	-1.277	1.124	0.903	H	-1.439586	1.494081	-0.503552
H	-0.012	-1.053	-0.903	H	-2.473959	2.836160	-0.999702
H	-0.011	-1.053	0.902	C	-3.303460	1.632247	0.610073
C	1.226	0.477	0.000	H	2.676171	0.689569	-1.259743
H	1.221	1.144	-0.903	C	4.123269	0.599668	0.356627
C	2.482	-0.367	0.000	H	3.633318	2.154242	-1.078372
H	1.221	1.144	0.902	H	3.594797	-0.180542	0.925073
H	2.488	-1.034	-0.903	H	4.552587	1.281262	1.106235
H	2.488	-1.034	0.902	C	5.260632	-0.046438	-0.445452
C	3.727	0.496	0.000	H	4.832854	-0.724536	-1.198692
H	3.719	1.162	-0.903	C	6.259610	-0.820124	0.424255
C	4.980	-0.341	0.000	H	5.795338	0.732509	-1.008878
H	3.718	1.163	0.902	H	5.722765	-1.598085	0.987666
H	5.018	-0.995	-0.904	H	6.686311	-0.141715	1.178155
H	5.017	-0.995	0.905	C	7.398091	-1.468879	-0.373898
O	-4.875	-0.433	0.000	H	6.971952	-2.145262	-1.129834
H	-5.665	0.119	-0.001	C	8.391766	-2.246458	0.498650
C	6.227	0.563	0.000	H	7.938133	-0.691224	-0.934397
H	6.220	1.180	0.874	H	7.850843	-3.024144	1.058471
H	6.220	1.180	-0.874	H	8.816565	-1.570053	1.255412
C	7.495	-0.312	0.000	C	9.532379	-2.896032	-0.295414
H	7.502	-0.930	-0.873	H	9.109338	-3.572607	-1.053105
H	8.361	0.316	0.000	H	10.075809	-2.119137	-0.853995
H	7.502	-0.930	0.874	C	-4.127798	0.690193	-0.277774
				H	-3.953166	2.430584	1.000880
				H	-2.917465	1.089251	1.482590
				H	-4.494764	1.240901	-1.156498
				C	-5.317112	0.052752	0.452574
				H	-3.473903	-0.103295	-0.667896
				H	-5.974090	0.846190	0.838668
				H	-4.950149	-0.493168	1.334050
				C	-6.135373	-0.899052	-0.429546
				H	-6.500156	-0.353129	-1.312305
				C	-7.325407	-1.537443	0.298497
				H	-5.477116	-1.691981	-0.814443
				H	-7.985310	-0.744467	0.680860
				H	-6.960626	-2.080456	1.183040
				C	-8.140583	-2.493592	-0.581965
				H	-8.503835	-1.951059	-1.467523
				C	-9.331814	-3.131241	0.144398
				H	-7.480502	-3.287087	-0.962898
				H	-9.993789	-2.338260	0.523614
				H	-8.969707	-3.672707	1.031197
				C	10.523144	-3.674445	0.579611
				H	10.944985	-2.998372	1.337112
				H	9.980409	-4.451774	1.136377
				C	11.661116	-4.318839	-0.218679
				H	12.349459	-4.866852	0.435062
				H	11.273414	-5.026596	-0.961871
				H	12.245183	-3.562461	-0.757522
				C	-10.145317	-4.089804	-0.734852
				H	-10.505866	-3.549335	-1.621708
				H	-9.484732	-4.883769	-1.111493
				C	-11.334639	-4.719885	-0.002733
				H	-11.892288	-5.400918	-0.655813
				H	-11.003001	-5.293681	0.871503
				H	-12.033441	-3.952361	0.352405

2-Heptanol **A3**							
Monomer				Dimer			
C	-3.764	0.450	0.000	H	0.373	2.997	-0.092
H	-3.796	1.104	0.913	O	1.123	2.648	-0.612
C	-2.512	-0.409	0.000	C	1.992	1.977	0.285
C	-1.272	0.458	0.000	C	3.123	1.342	-0.517
H	-2.521	-1.074	-0.902	H	1.458	1.194	0.852
H	-2.521	-1.074	0.902	H	-1.817	3.860	0.945
H	-1.277	1.125	-0.902	O	-1.316	3.061	0.724
C	-0.016	-0.387	0.000	C	-2.126	2.239	-0.130
H	-1.277	1.124	0.903	H	-1.443	1.471	-0.505
H	-0.012	-1.053	-0.903	C	-3.306	1.615	0.609
H	-0.011	-1.053	0.902	H	2.682	0.667	-1.263
O	-4.875	-0.433	0.000	C	4.128	0.580	0.355
H	-5.665	0.119	-0.001	H	3.636	2.134	-1.080
C	1.248	0.492	0.000	H	3.601	-0.202	0.922
H	1.254	1.109	-0.875	H	4.554	1.262	1.106
H	1.252	1.112	0.873	C	5.269	-0.061	-0.446
C	2.499	-0.406	0.003	H	4.844	-0.741	-1.200
H	2.495	-1.026	-0.870	C	6.269	-0.832	0.425
H	3.377	0.205	0.002	H	5.801	0.720	-1.008
H	2.493	-1.022	0.878	H	5.735	-1.613	0.986
C	-3.808	1.346	-1.251	H	6.692	-0.152	1.180
H	-3.179	2.199	-1.102	C	-4.134	0.676	-0.278
H	-3.462	0.793	-2.099	H	-3.953	2.415	1.001
H	-4.813	1.670	-1.423	H	-2.921	1.070	1.481
				H	-4.500	1.228	-1.156
				C	-5.325	0.042	0.453
				H	-3.483	-0.120	-0.669
				H	-5.978	0.837	0.840
				H	-4.959	-0.507	1.333
				C	-6.147	-0.906	-0.430
				H	-6.511	-0.358	-1.311
				H	-5.492	-1.701	-0.816
				C	-7.343	-1.544	0.302
				H	-8.002	-0.774	0.646
				H	-7.869	-2.189	-0.370
				H	-6.990	-2.111	1.137
				C	7.416	-1.477	-0.375
				H	8.071	-1.994	0.295
				H	7.012	-2.169	-1.084
				H	7.963	-0.716	-0.892
				C	2.583	2.948	1.324
				H	2.950	2.393	2.163
				H	3.388	3.497	0.881
				H	1.824	3.627	1.650
				C	-2.613	3.046	-1.348
				H	-2.827	2.378	-2.156
				H	-3.499	3.586	-1.087
				H	-1.850	3.734	-1.647

5-Nonanol **A4**							
Monomer				Dimer			
C	-3.764	0.450	0.000	H	0.309234	2.434278	0.106119
H	-3.796	1.104	0.913	O	1.124640	2.441793	-0.435009
H	-3.796	1.104	-0.913	C	2.121692	1.666554	0.226875
C	-2.512	-0.409	0.000	C	1.606806	0.241264	0.497179
C	-1.272	0.458	0.000	H	2.369222	2.132354	1.199384
H	-2.521	-1.074	-0.902	H	-1.801004	3.325769	0.615759
H	-2.521	-1.074	0.902	O	-1.484754	2.412108	0.717416
H	-1.277	1.125	-0.902	C	-2.280785	1.581095	-0.161512
C	-0.016	-0.387	0.000	H	-1.833113	0.586208	-0.045588
H	-1.277	1.124	0.903	C	-3.733190	1.538468	0.326333
H	-0.012	-1.053	-0.903	H	0.642204	0.331195	1.018916
C	1.226	0.477	0.000	C	2.534307	-0.647469	1.337095
H	1.221	1.144	-0.903	H	1.393961	-0.239965	-0.468290
C	2.482	-0.367	0.000	H	2.809764	-0.117636	2.261673
H	1.221	1.144	0.902	H	3.474460	-0.828221	0.798277
H	2.488	-1.034	-0.903	C	1.905176	-1.998959	1.702856
H	2.488	-1.034	0.902	H	0.970562	-1.824659	2.254823
C	3.727	0.496	0.000	H	1.619641	-2.526262	0.781398
H	3.719	1.162	-0.903	C	-3.891612	1.019404	1.760937
C	4.980	-0.341	0.000	H	-4.318500	0.909492	-0.357366
H	3.718	1.163	0.902	H	-4.160618	2.551605	0.245937
H	5.018	-0.995	-0.904	H	-3.486126	-0.001768	1.820053
H	5.017	-0.995	0.905	C	-5.345833	1.014531	2.248722
H	5.884	0.314	0.000	H	-3.276414	1.629020	2.434325
O	-0.010	-1.236	1.150	H	-5.957577	0.405540	1.567628
H	0.002	-2.155	0.871	H	-5.752115	2.034687	2.189996
C	-4.970	-0.508	0.000	C	3.371058	1.706957	-0.656244
H	-5.876	0.059	-0.039	H	4.152077	1.082101	-0.202971
H	-4.914	-1.151	-0.853	H	3.118256	1.248825	-1.623640
H	-4.956	-1.098	0.893	C	-2.106629	2.038078	-1.616381
				H	-2.548695	3.041389	-1.730449
				H	-1.029302	2.143774	-1.793667
				C	3.919177	3.121186	-0.883774
				H	3.127526	3.742833	-1.318281
				H	4.168428	3.573910	0.088258
				C	5.159062	3.151747	-1.786191
				H	5.945911	2.519242	-1.349645
				H	4.911230	2.699382	-2.757199
				C	5.707648	4.564989	-2.008594
				H	5.997117	5.031329	-1.058503
				H	6.590859	4.556858	-2.658067
				H	4.955125	5.211737	-2.476454
				C	2.831268	-2.891239	2.535520
				H	3.108940	-2.403397	3.478106
				H	2.352618	-3.845749	2.783202
				H	3.758960	-3.114619	1.994187
				C	-2.704372	1.094871	-2.670364
				H	-2.311371	0.079232	-2.512803
				H	-3.793929	1.024185	-2.547729
				C	-2.393090	1.535814	-4.107304
				H	-2.778878	2.553103	-4.264716
				H	-1.304023	1.600552	-4.236401
				C	-2.980890	0.597073	-5.165571
				H	-2.741175	0.938174	-6.179070
				H	-2.587026	-0.420818	-5.055250
				H	-4.073428	0.539304	-5.083533
				C	-5.497424	0.487960	3.679385
				H	-6.544987	0.499166	4.001275
				H	-5.134255	-0.543807	3.762536
				H	-4.923817	1.097433	4.388396

2,4-Dimethyl-4-heptanol **A5**							
Monomer				Dimer			
C	-2.512	-0.409	0.000	C	2.267690	2.208619	0.532389
C	-1.272	0.458	0.000	H	2.338816	2.640858	1.543342
H	-2.521	-1.074	0.902	C	3.501955	2.625373	-0.272650
H	-1.277	1.125	-0.902	H	4.402503	2.268674	0.247489
C	-0.016	-0.387	0.000	C	3.639874	4.138477	-0.534914
H	-1.277	1.124	0.903	H	3.469029	2.101924	-1.239200
H	-0.012	-1.053	-0.903	H	2.736499	4.457222	-1.069198
C	1.226	0.477	0.000	O	1.128704	2.755563	-0.134502
H	1.221	1.144	-0.903	H	0.331810	2.578073	0.404680
C	2.482	-0.367	0.000	C	4.853312	4.410116	-1.436455
H	1.221	1.144	0.902	H	4.937001	5.476357	-1.679825
H	2.488	-1.034	0.902	H	5.788121	4.108862	-0.944412
O	-0.010	-1.236	1.150	H	4.782515	3.856045	-2.380482
H	0.002	-2.155	0.871	C	3.732596	4.957223	0.760971
C	-3.782	0.462	0.000	H	2.827008	4.856244	1.368353
H	-3.790	1.080	-0.874	H	4.588398	4.639560	1.372551
H	-4.647	-0.168	0.000	H	3.864159	6.023358	0.539406
H	-3.791	1.080	0.874	C	-1.834043	4.026894	-1.592254
C	3.748	0.510	0.000	C	-1.988329	2.521517	-1.292806
H	4.615	-0.116	0.000	H	-1.116194	4.443167	-0.869543
H	3.753	1.128	-0.874	H	-2.732100	2.094145	-1.981644
H	3.753	1.128	0.874	C	-2.399361	2.159866	0.142454
C	2.490	-1.282	-1.239	H	-1.031390	2.027375	-1.501715
H	1.953	-2.181	-1.021	H	-3.378995	2.602472	0.370992
H	2.024	-0.777	-2.058	C	-2.467816	0.646116	0.350889
H	3.500	-1.524	-1.497	H	-3.168872	0.233845	-0.389451
C	-2.524	-1.323	-1.240	C	-2.879036	0.177315	1.760896
H	-2.066	-0.815	-2.062	H	-1.480255	0.229187	0.108374
H	-1.981	-2.220	-1.027	H	-2.185808	0.639236	2.475628
H	-3.535	-1.569	-1.491	O	-1.443031	2.667863	1.104656
				H	-1.507954	3.635878	1.105039
				C	-1.215695	4.223595	-2.984030
				H	-1.859312	3.796929	-3.764810
				H	-1.079887	5.287972	-3.209532
				H	-0.236605	3.736474	-3.045470
				C	-2.732826	-1.347783	1.870526
				H	-2.980031	-1.697968	2.879757
				H	-3.403765	-1.860377	1.168268
				H	-1.708632	-1.669320	1.646254
				C	-4.304788	0.610385	2.134600
				H	-4.413873	1.700405	2.152888
				H	-5.038847	0.210737	1.421825
				H	-4.575317	0.239339	3.130154
				C	-3.157804	4.797776	-1.468567
				H	-3.900697	4.413072	-2.179566
				H	-3.596632	4.726426	-0.465773
				H	-3.012708	5.862561	-1.685628
				C	3.226258	-0.095701	1.385656
				H	4.175956	0.086688	0.862200
				C	2.947739	-1.605341	1.316876
				H	2.007549	-1.854478	1.826734
				H	2.864186	-1.949675	0.279151
				H	3.748472	-2.179459	1.798480
				C	3.397181	0.352122	2.846100
				H	4.169835	-0.241867	3.348969
				H	3.687313	1.405471	2.925286
				H	2.461609	0.221409	3.406154
				C	2.112842	0.680573	0.653671
				H	2.000860	0.282582	-0.364794
				H	1.158810	0.482030	1.167561

Menthol **A6**							
Monomer				Dimer			
C	-3.323	-1.241	0.000	C	4.103936	1.743444	-1.996220
C	-1.808	-1.241	0.000	C	2.973847	0.751234	-2.351339
C	-1.256	0.170	0.000	C	2.176599	0.378587	-1.079725
C	-1.806	0.975	1.161	C	1.734242	1.567120	-0.212511
C	-3.321	0.975	1.160	C	2.917794	2.509692	0.143185
C	-3.874	-0.435	1.159	C	3.600204	2.941000	-1.174587
H	-0.137	0.136	0.063	H	1.284149	-0.200205	-1.348838
H	-1.433	-1.791	-0.902	H	3.449246	-0.172763	-2.712755
H	-3.696	-0.808	-0.965	H	4.883759	1.212732	-1.430908
H	-3.699	-2.295	0.064	H	4.585664	2.099780	-2.916849
H	-1.430	2.029	1.099	H	1.296394	1.161392	0.710468
H	-3.696	1.525	2.062	H	2.474887	3.411379	0.598422
H	-3.608	-0.941	2.124	H	2.860112	3.496166	-1.761608
H	-4.992	-0.400	1.094	H	4.421963	3.638291	-0.978121
H	-1.525	0.676	-0.965	H	2.797975	-0.277845	-0.454881
C	-1.297	-1.990	1.245	C	2.081083	1.264385	-3.494186
H	-1.667	-2.994	1.234	H	2.681359	1.463033	-4.391423
H	-0.227	-2.004	1.238	H	1.325423	0.514199	-3.757597
H	-1.641	-1.492	2.127	H	1.548285	2.176698	-3.217229
C	-3.831	1.726	-0.084	C	3.881028	1.915266	1.212873
H	-3.493	1.224	-0.967	H	4.219341	0.925745	0.874276
C	-3.289	3.167	-0.073	C	3.179976	1.724305	2.570996
H	-2.361	3.203	-0.605	H	2.326613	1.041637	2.517002
H	-3.995	3.818	-0.544	H	3.878254	1.316790	3.311771
H	-3.133	3.482	0.938	H	2.813381	2.684912	2.957218
C	-5.371	1.757	-0.069	C	5.136487	2.781346	1.417557
H	-5.716	1.846	0.940	H	4.865491	3.814039	1.675510
H	-5.716	2.593	-0.640	H	5.745546	2.385651	2.239125
H	-5.750	0.852	-0.497	H	5.771061	2.815518	0.526812
O	-1.331	0.420	2.390	O	0.720200	2.283340	-0.921926
H	-0.765	1.058	2.832	H	0.263241	2.869357	-0.285063
				C	-4.057536	2.254883	1.891188
				C	-3.085257	1.529659	0.934343
				C	-2.672596	2.467662	-0.224993
				C	-2.250047	3.882777	0.188571
				C	-3.261804	4.570634	1.137454
				C	-3.527834	3.628184	2.333373
				H	-1.857363	2.020995	-0.805448
				H	-3.632237	0.691572	0.479096
				H	-5.029050	2.380882	1.392705
				H	-4.245707	1.628358	2.772905
				H	-2.119179	4.479218	-0.723059
				H	-2.770509	5.479578	1.528128
				H	-2.584338	3.495024	2.875045
				H	-4.220334	4.092810	3.042567
				H	-3.522802	2.577812	-0.911532
				C	-1.883224	0.919137	1.675013
				H	-2.221775	0.189434	2.421223
				H	-1.222471	0.398573	0.972208
				H	-1.284129	1.678794	2.184049
				C	-4.543172	5.066086	0.402186
				H	-5.007528	4.211915	-0.109655
				C	-4.221764	6.128391	-0.666149
				H	-3.582581	5.748213	-1.468695
				H	-5.144061	6.492246	-1.133427
				H	-3.717054	6.994619	-0.217602
				C	-5.585294	5.637787	1.379391
				H	-5.155886	6.446507	1.985528
				H	-6.435397	6.054813	0.827440
				H	-5.979761	4.879822	2.062536
				O	-0.954436	3.783397	0.832873
				H	-0.677413	4.681995	1.074289

4-Pentylcyclohexanol **A7**							
Monomer				Dimer			
H	0.372	2.995	-0.092	H	0.767409	1.674088	-0.418660
O	1.123	2.648	-0.612	O	1.445803	1.083554	-0.805621
C	2.000	1.971	0.293	H	-0.672729	3.548067	-0.377708
C	2.107	0.512	-0.100	O	-0.790585	2.657738	-0.008417
C	3.358	2.644	0.283	C	2.245893	0.564979	0.249913
H	1.552	2.050	1.318	C	3.241373	-0.419753	-0.361619
C	3.092	-0.227	0.783	C	2.986168	1.673534	1.011040
H	2.437	0.437	-1.169	H	1.612781	0.014991	0.970905
H	1.099	0.028	-0.024	C	4.197073	-0.999822	0.691890
C	4.342	1.906	1.167	H	3.812548	0.112458	-1.135475
H	3.750	2.675	-0.767	H	2.692005	-1.220774	-0.871171
H	3.255	3.702	0.637	C	3.937581	1.096805	2.070475
C	4.449	0.446	0.776	H	3.550886	2.268335	0.279236
H	3.195	-1.285	0.428	H	2.258156	2.350460	1.479252
H	2.698	-0.260	1.833	C	4.947701	0.097882	1.472322
H	5.351	2.390	1.091	H	4.906911	-1.676940	0.202193
H	4.012	1.982	2.236	H	3.627138	-1.613780	1.407058
H	4.899	0.366	-0.249	H	4.473040	1.910575	2.577371
C	5.391	-0.282	1.753	H	3.350366	0.584016	2.848113
H	5.482	-1.308	1.463	H	5.573440	0.650310	0.751619
H	4.991	-0.225	2.743	C	-1.882103	2.031315	-0.713813
C	6.779	0.385	1.720	C	-1.666522	2.052175	-2.229460
H	6.775	1.248	2.353	C	-1.992186	0.598613	-0.199683
H	7.010	0.679	0.718	H	-2.810686	2.574174	-0.470683
C	9.207	0.090	2.304	C	-2.782758	1.292497	-2.965542
H	9.220	0.747	3.148	H	-0.692478	1.592433	-2.442791
H	9.373	0.655	1.410	H	-1.619041	3.091398	-2.587631
C	10.319	-0.965	2.457	C	-3.106533	-0.164600	-0.931008
H	11.105	-0.758	1.761	H	-1.024838	0.106687	-0.365350
H	10.707	-0.932	3.454	H	-2.171985	0.610064	0.882078
H	9.917	-1.937	2.263	C	-2.924084	-0.157718	-2.461756
C	7.840	-0.613	2.220	H	-2.579960	1.309078	-4.042375
H	7.860	-1.431	1.531	H	-3.741584	1.815170	-2.821824
H	7.606	-0.977	3.199	H	-3.144457	-1.198741	-0.565586
				H	-4.081608	0.284445	-0.685755
				H	-1.979784	-0.680310	-2.685266
				C	5.873199	-0.467635	2.565888
				H	6.288404	0.374026	3.140319
				H	5.269088	-1.049061	3.279509
				C	7.035715	-1.335636	2.063440
				H	6.646748	-2.217450	1.536413
				H	7.618295	-0.769255	1.321240
				C	7.968129	-1.801197	3.189794
				H	7.383907	-2.358529	3.937455
				H	8.371078	-0.922507	3.715606
				C	9.131050	-2.676202	2.704311
				H	8.728312	-3.554359	2.179607
				H	9.716122	-2.119490	1.958352
				C	10.054549	-3.137849	3.836397
				H	10.873901	-3.759643	3.457595
				H	10.500017	-2.282010	4.358627
				H	9.505031	-3.727981	4.580241
				C	-4.065689	-0.926793	-3.151839
				H	-4.159566	-1.910942	-2.669240
				H	-5.016918	-0.406343	-2.960401
				C	-3.898605	-1.138457	-4.663383
				H	-3.884512	-0.169131	-5.180417
				H	-2.920509	-1.602910	-4.859160
				C	-5.003456	-2.011405	-5.273541
				H	-5.010777	-2.990880	-4.772206
				H	-5.983751	-1.557412	-5.063970
				C	-4.857721	-2.220068	-6.786321
				H	-4.858841	-1.242062	-7.288475
				H	-3.876203	-2.668140	-6.997140
				C	-5.959679	-3.101150	-7.383699
				H	-6.951613	-2.662813	-7.217732
				H	-5.829062	-3.228962	-8.464403
				H	-5.959992	-4.099156	-6.928140

2-methyl-2-hexanol **A8**							
Monomer				Dimer			
C	-3.764	0.450	0.000	H	0.372	2.995	-0.092
C	-2.512	-0.409	0.000	O	1.123	2.648	-0.612
C	-1.272	0.458	0.000	H	-1.817	3.861	0.944
H	-2.521	-1.074	-0.902	O	-1.316	3.061	0.724
H	-2.521	-1.074	0.902	C	2.000	1.971	0.292
H	-1.277	1.125	-0.902	C	-2.123	2.242	-0.127
C	-0.016	-0.387	0.000	C	3.203	1.405	-0.486
H	-1.277	1.124	0.903	H	2.855	0.717	-1.228
H	-0.012	-1.053	-0.903	H	3.729	2.206	-0.961
H	-0.011	-1.053	0.902	C	-2.464	3.019	-1.412
O	-4.875	-0.433	0.000	H	-2.999	2.380	-2.084
H	-5.665	0.119	-0.001	H	-3.070	3.866	-1.167
C	-3.808	1.346	-1.251	H	-1.560	3.351	-1.879
H	-3.179	2.199	-1.102	C	2.499	2.961	1.361
H	-3.462	0.793	-2.099	H	1.668	3.316	1.934
H	-4.813	1.670	-1.423	H	3.193	2.467	2.008
C	1.248	0.492	0.000	H	2.983	3.787	0.884
H	1.230	1.144	0.849	C	-1.349	0.961	-0.491
H	2.116	-0.131	0.048	H	-1.104	0.426	0.403
H	1.277	1.075	-0.897	H	-1.956	0.345	-1.121
C	-3.808	1.346	1.251	H	-0.449	1.223	-1.008
H	-4.801	1.722	1.387	C	-3.424	1.865	0.606
H	-3.523	0.775	2.110	H	-3.185	1.349	1.513
H	-3.131	2.166	1.127	H	-3.975	2.753	0.835
				C	1.242	0.818	0.975
				H	0.868	0.146	0.231
				H	1.907	0.292	1.628
				H	0.425	1.214	1.541
				C	4.147	0.676	0.488
				H	4.496	1.364	1.229
				H	3.619	-0.124	0.965
				C	5.347	0.106	-0.290
				H	4.998	-0.583	-1.031
				H	5.875	0.905	-0.768
				C	6.292	-0.623	0.684
				H	7.126	-1.019	0.143
				H	6.641	0.066	1.424
				H	5.764	-1.422	1.161
				C	-4.275	0.951	-0.295
				H	-3.768	0.019	-0.438
				H	-4.425	1.424	-1.243
				C	-5.639	0.697	0.373
				H	-5.488	0.248	1.333
				H	-6.157	1.626	0.491
				C	-6.475	-0.249	-0.509
				H	-5.954	-1.176	-0.631
				H	-7.420	-0.429	-0.043
				H	-6.630	0.201	-1.468

3-Ethyl-3-pentanol **A9**							
Monomer				Dimer			
C	-0.012	-0.336	-0.023	C	-2.524	-0.052	-0.322
C	0.008	0.947	-0.874	C	-2.165	0.167	1.159
H	0.864	0.935	-1.516	H	-1.391	-0.515	1.442
H	-0.881	0.997	-1.466	H	-1.825	1.171	1.299
C	-1.270	-0.343	0.866	C	-3.603	0.963	-0.745
H	-1.284	-1.234	1.457	H	-3.853	0.811	-1.773
H	-1.255	0.512	1.510	H	-4.477	0.825	-0.142
C	0.078	2.176	0.052	C	-3.411	-0.079	2.031
H	0.509	2.999	-0.479	H	-3.107	-0.348	3.021
H	-0.908	2.436	0.374	H	-4.001	0.813	2.069
H	0.683	1.946	0.904	H	-3.991	-0.873	1.608
C	-2.527	-0.295	-0.022	C	-3.067	2.394	-0.551
H	-3.359	-0.700	0.516	H	-3.601	3.064	-1.192
H	-2.735	0.719	-0.291	H	-3.201	2.690	0.468
H	-2.360	-0.872	-0.908	H	-2.025	2.420	-0.796
C	2.502	-0.380	-0.024	C	-1.981	-2.496	-0.096
H	2.286	-0.873	-0.949	H	-1.011	-2.086	-0.290
H	2.799	0.629	-0.219	H	-2.075	-2.708	0.948
H	3.295	-0.895	0.477	H	-2.103	-3.400	-0.656
C	1.245	-0.384	0.865	C	-3.060	-1.482	-0.516
H	1.158	-1.270	1.458	H	-3.329	-1.563	-1.548
H	1.332	0.468	1.508	H	-3.917	-1.690	0.093
O	-0.031	-1.478	-0.883	O	-1.357	0.132	-1.128
H	-0.047	-2.279	-0.353	H	-1.581	-0.001	-2.052
				C	2.320	0.074	-0.025
				C	3.428	-0.088	1.032
				H	3.362	0.709	1.742
				H	3.308	-1.024	1.535
				C	2.383	-1.101	-1.020
				H	1.614	-0.988	-1.754
				H	3.339	-1.107	-1.502
				C	4.805	-0.049	0.343
				H	5.548	0.263	1.047
				H	5.048	-1.024	-0.024
				H	4.775	0.642	-0.473
				C	2.182	-2.427	-0.263
				H	1.822	-3.173	-0.940
				H	3.114	-2.744	0.156
				H	1.469	-2.284	0.523
				C	2.455	2.573	0.212
				H	1.790	2.325	1.013
				H	3.431	2.764	0.606
				H	2.096	3.447	-0.291
				C	2.521	1.399	-0.782
				H	1.752	1.439	-1.524
				H	3.479	1.465	-1.257
				O	1.044	0.083	0.620
				H	0.352	0.180	-0.039

1,1,1-Trifluoro-2-octanol **A10**							
Monomer				Dimer			
C	-3.764	0.450	0.000	H	0.798794	1.497284	-1.177693
C	-2.512	-0.409	0.000	O	1.643826	1.980467	-1.267453
C	-1.272	0.458	0.000	C	1.664179	3.023891	-0.318652
H	-2.521	-1.074	-0.902	C	3.098249	3.559163	-0.257010
H	-1.277	1.125	-0.902	H	1.350122	2.674530	0.676380
C	-0.016	-0.387	0.000	H	-0.394634	-0.528250	-0.452998
H	-1.277	1.124	0.903	O	-0.650351	0.324359	-0.843159
H	-0.012	-1.053	-0.903	C	-1.933564	0.683497	-0.329573
H	-0.011	-1.053	0.902	H	-2.145660	1.671328	-0.744147
C	1.226	0.477	0.000	C	-3.001553	-0.330720	-0.760923
H	1.221	1.144	-0.903	H	3.732460	2.677192	-0.106593
C	2.482	-0.367	0.000	C	3.390494	4.598099	0.834087
H	1.221	1.144	0.902	H	3.354423	3.959212	-1.245502
H	2.488	-1.034	-0.903	H	3.066115	4.207710	1.809964
H	2.488	-1.034	0.902	H	2.802626	5.506658	0.659879
C	3.727	0.496	0.000	C	4.878592	4.965591	0.905242
H	3.719	1.162	-0.903	H	5.470784	4.056754	1.088879
C	4.980	-0.341	0.000	C	5.197200	6.006088	1.986226
H	3.718	1.163	0.902	H	5.204826	5.347188	-0.073602
H	5.018	-0.995	-0.904	H	4.874963	5.623040	2.966161
H	5.017	-0.995	0.905	H	4.600654	6.912589	1.804827
H	5.884	0.314	0.000	C	6.682187	6.384547	2.055259
O	-2.523	-1.258	1.151	H	7.279058	5.479037	2.235636
H	-2.880	-2.116	0.912	H	7.003071	6.769325	1.076741
F	-4.821	-0.390	0.000	C	-4.463953	0.109042	-0.589660
F	-3.802	1.236	1.097	H	-2.829144	-1.278148	-0.230902
F	-3.802	1.236	-1.097	H	-2.803967	-0.524905	-1.822155
				H	-4.697077	0.240884	0.471964
				C	-5.448287	-0.893334	-1.207262
				H	-4.603758	1.092636	-1.061008
				H	-5.293153	-1.882711	-0.751855
				H	-5.224428	-1.012539	-2.277547
				C	-6.916130	-0.482025	-1.036868
				H	-7.138359	-0.364792	0.034110
				C	-7.906262	-1.478916	-1.652845
				H	-7.071228	0.508947	-1.488845
				H	-7.749128	-2.469728	-1.203181
				H	-7.685768	-1.593002	-2.723733
				C	0.641879	4.094139	-0.718355
				C	-1.803090	0.849371	1.185864
				C	6.991575	7.423008	3.138468
				H	6.713387	7.052963	4.133105
				H	8.058785	7.671739	3.162401
				H	6.436516	8.353417	2.965925
				C	-9.369914	-1.063188	-1.474142
				H	-9.566053	-0.091042	-1.942965
				H	-10.051087	-1.793634	-1.925139
				H	-9.630560	-0.976028	-0.412167
				F	-0.571790	3.530880	-0.970457
				F	0.448143	5.002298	0.267042
				F	0.997458	4.769248	-1.827209
				F	-0.872940	1.769998	1.506847
				F	-2.959043	1.226161	1.766077
				F	-1.418487	-0.323317	1.754517
